# Pathological and neurophysiological outcomes of seeding human-derived tau pathology in the APP-KI NL-G-F and NL-NL mouse models of Alzheimer’s Disease

**DOI:** 10.1186/s40478-022-01393-w

**Published:** 2022-06-23

**Authors:** S. Tok, H. Maurin, C. Delay, D. Crauwels, N. V. Manyakov, W. Van Der Elst, D. Moechars, W. H. I. M. Drinkenburg

**Affiliations:** 1 Department of Neuroscience, Janssen Research and Development, Janssen Pharmaceutica NV, Turnhoutseweg 30, 2340 Beerse, Belgium; 2 Data Sciences, Janssen Research and Development, Janssen Pharmaceutica NV, Turnhoutseweg 30, 2340 Beerse, Belgium; 3 Quantitative Sciences Janssen Research and Development, Janssen Pharmaceutica NV, Turnhoutseweg 30, 2340 Beerse, Belgium; 4 Groningen Institute for Evolutionary Life Sciences, Faculty of Science and Engineering, University of Groningen, Groningen, The Netherlands

## Abstract

The two main histopathological hallmarks that characterize Alzheimer’s Disease are the presence of amyloid plaques and neurofibrillary tangles. One of the current approaches to studying the consequences of amyloid pathology relies on the usage of transgenic animal models that incorporate the mutant humanized form of the amyloid precursor protein (hAPP), with animal models progressively developing amyloid pathology as they age. However, these mice models generally overexpress the hAPP protein to facilitate the development of amyloid pathology, which has been suggested to elicit pathological and neuropathological changes unrelated to amyloid pathology. In this current study, we characterized APP knock-in (APP-KI) animals, that do not overexpress hAPP but still develop amyloid pathology to understand the influence of protein overexpression. We also induced tau pathology via human-derived tau seeding material to understand the neurophysiological effects of amyloid and tau pathology. We report that tau-seeded APP-KI animals progressively develop tau pathology, exacerbated by the presence of amyloid pathology. Interestingly, older amyloid-bearing, tau-seeded animals exhibited more amyloid pathology in the entorhinal area, isocortex and hippocampus, but not thalamus, which appeared to correlate with impairments in gamma oscillations before seeding. Tau-seeded animals also featured immediate deficits in power spectra values and phase-amplitude indices in the hippocampus after seeding, with gamma power spectra deficits persisting in younger animals. Both deficits in hippocampal phase-amplitude coupling and gamma power differentiate tau-seeded, amyloid-positive animals from buffer controls. Based on our results, impairments in gamma oscillations appear to be strongly associated with the presence and development of amyloid and tau pathology, and may also be an indicator of neuropathology, network dysfunction, and even potential disposition to the future development of amyloid pathology.

**Supplementary Information:**

The online version contains supplementary material available at 10.1186/s40478-022-01393-w.

## Introduction

The progressive cognitive impairment and decline in patients suffering from Alzheimer’s Disease (AD) is believed to arise from the accumulation of amyloid-beta (Aβ) in the form of amyloid plaques and tau neurofibrillary tangles (NFTs) [1, 2] and is thought to characterize AD. Approaches to studying the effects of both amyloid and tau pathology have involved the usage of animal models that mimic or exhibit certain aspects of AD-associated pathology [3]. These transgenic animal models generally involve the expression of mutant forms of the human protein associated with the development of AD-associated pathologies, such as the amyloid precursor protein (APP) containing the Swedish mutation and Presenilin mutations in the APP.PS1/L166P amyloid mouse model [4], or the PS19 mouse model that expresses the P301S tau mutation [5], which results in the progressive development of amyloid plaque and tau NFT pathology respectively.

The expression of these transgenes is usually driven by an endogenous mouse, neuron-specific [6, 7] promoter (e.g., Thy1 or PDGF promoters). However, protein expression under these promoters has been reported to be relatively high, with reports of up to 15-fold above endogenous levels in the case of the PrP promoter [3]. As such, levels of Aβ_40_ and Aβ_42_ [8], which are believed to underlie the formation of amyloid plaque pathology increase drastically and are believed to promote the development of amyloid plaque pathology. However, in addition to increases in Aβ_40_ and Aβ_42_, other cleavage products such as the APP intracellular domain (AICD), C-terminal fragment α/β, soluble APP α/β, p3[9], and Aη [10], are some of the other currently identified cleavage products likely to increase as APP expression levels also increase.

These other fragments have been shown to exhibit neurophysiological capabilities in modulating long-term potentiation (LTP)[11], reducing neuronal activity [10], and altering synaptic properties [12], among numerous other effects [13]. Furthermore, even just the overexpression of wild-type APP in mice has also been related to deficits in cognition and pathological features believed to be unrelated to Aβ levels [14]. Thus, it becomes hard to discern the neurophysiological effects purely associated with amyloid pathology from those of APP fragments or of the expression of APP itself in these animal models.

Newer animal models, such as the APP-knock-in (APP-KI) mouse model family, aim to address some of these limitations by replacing the endogenous mouse APP gene with a mutant humanized form of the APP gene (i.e., knocking in). This results in comparable expression levels of APP while still retaining the development of amyloid pathology. The genes encoding for APP inserted into these mice contain one or more mutations that shift the balance of the APP processing pathway towards the formation of Aβ_40_ and Aβ_42_ fragments [15]. The APP-KI mouse model family contains mice that exhibit heterozygous and homozygous combinations of 3 mutations associated with amyloid pathology development: the Swedish (NL) [16], Arctic (G) [17] and Beyreuther/Iberian (F) mutations [18, 19] on the APP transgene. These mutations exhibit an additive effect, with the combination of all 3 mutations (i.e., APP-KI^NL-G-F^) exhibiting earlier and more aggressive amyloid deposition compared to mice that possess two or only one of these mutations (i.e., APP-KI^NL−F^ and APP-KI^NL^) mice.

In terms of tau pathology, transgenic animal models have also been used to study tau pathology. However, these animal models derive tau pathology via mutations in the microtubule associated protein tau (MAPT) gene, which is not representative of AD [20]. Another recent approach to inducing tau pathology involves the injection of material that promotes the conformation of endogenous tau into pathological species, a process termed “tau seeding”. Seeding material derived from the purification of diseased human brain tissue is thought to represent the physiological processes underlying tau aggregation and spreading in AD moreso than mutations [21].

## Methods and materials

### Aim and design of the study

In this present study, we sought to evaluate the influence of tau seeding in APP-KI animals in terms of longitudinal pathological and neurophysiological changes and determine if and which neurophysiological changes could be attributed to the presence of amyloid pathology, in the absence of confounds associated with protein overexpression. APP-KI^NL-NL^ animals were selected as the control animals for APP-KI^NL-G-F^ animals in this study due to the absence of amyloid plaque development at the ages selected for this study. This allowed for drawing conclusions linked to the presence of amyloid plaque pathology and mutations on the APP protein, while still controlling for the insertional effects associated with the knock-in protein, if any. This study was framed as an exploratory study aimed at characterizing and understanding the changes associated with both amyloid and tau pathology in this animal model. The tau seeding approach in this study derives tau pathology from the injection of seeding material obtained from purified human brain extracts, which may be more representative of tau pathology seen in AD [21], as opposed to transgenic mouse models of tau which derive pathology from mutations associated with frontotemporal dementia. However, it should still be noted that mice and humans still differ significantly in terms of endogenous tau properties and mice still do not fully capitulate all the nuances of tau pathology in humans.

One primary goal in the field of AD is the early detection and staging of the disease, to provide therapeutic intervention before irreversible neurophysiological changes occur. As such, indicators or biomarkers for the early stages of the disease are highly sought after, especially if these biomarkers are non-invasive and robust. Electroencephalography (EEG) is one promising approach for the detection of neurophysiological changes associated with the progression of AD, even at the early stages of the disease [22–24], and was thus chosen as a tool to evaluate and detect functional changes in this present study from the incipience of pathology.

Determining AD-relevant neurophysiological changes in the absence of protein overexpression confounds provides a clearer understanding of pathology-specific changes. A clearer understanding of pathology-related changes allows for the potential elucidation of disease-specific biomarkers, as well as providing insight into relevant pathways associated with neurophysiological changes, which may be clinically relevant for the diagnosis and staging, as well as detection of AD using EEG and related methods.

### Animal cohorts and usage

Data were obtained from homozygous APP^NL-G-F/NL-G-F^ and APP^NL/NL^ mice on the C57BL/6 background (Breeding done in Transgenic Rodent Facility, Janssen Pharmaceutica, original breeding pair obtained from Takaomi Saido, RIKEN). Animal housing conditions, diet, and light–dark cycles, except for food restriction were identical to the conditions listed in (Jacob et al., 2019). Animals were given ad libitum access to food (SAFE A05 diet, SAFE DIETS) and water. Animals were genotyped using PCR of ear punches.

**Fig. 1:**
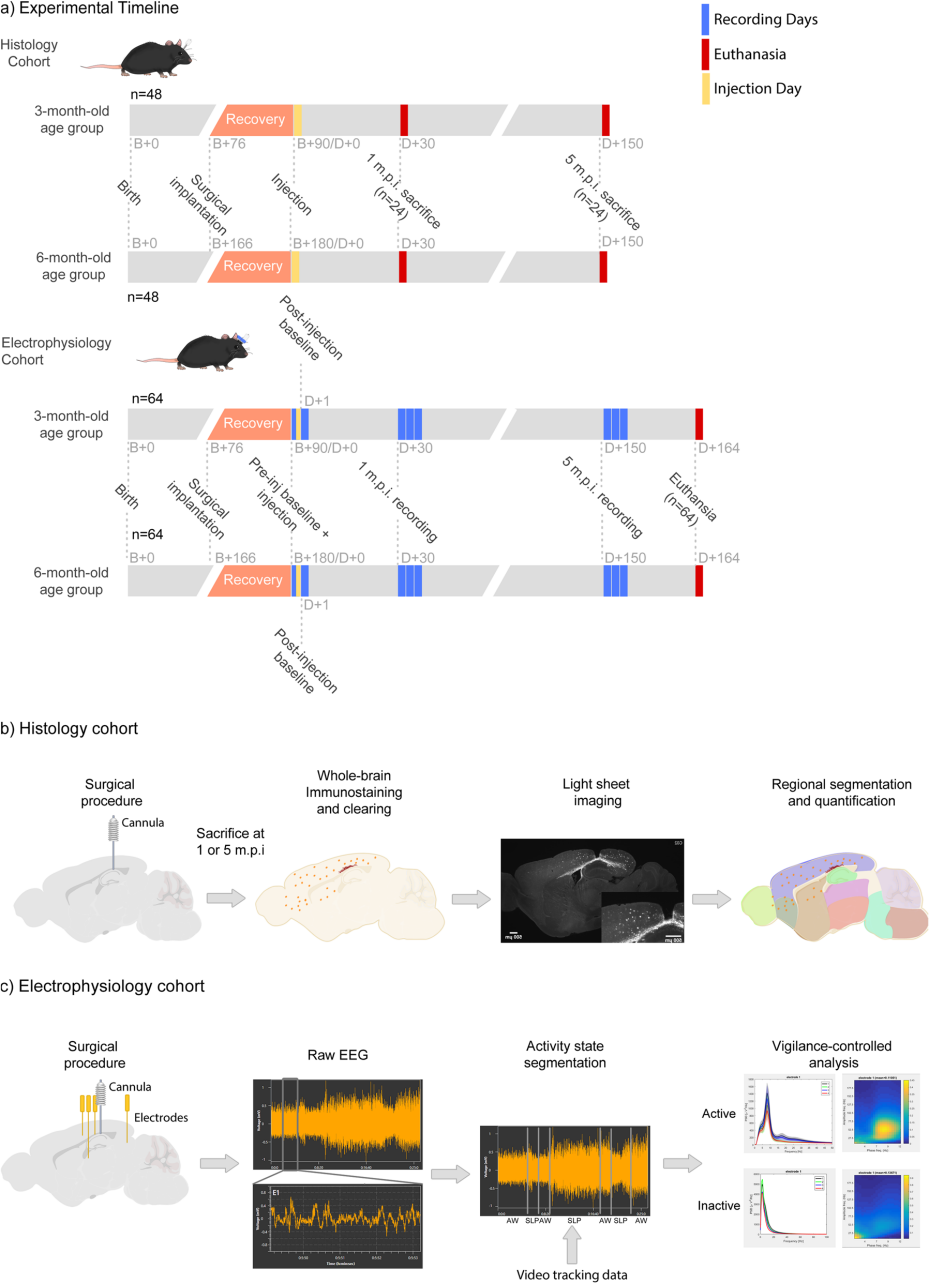
Illustrated overview of the experimental study, containing the workflow for histological and electrophysiological assessment. **a** Timeline for both histological and electrophysiological cohorts indicating sample sizes, recording, euthanasia and injection timepoints for two age groups. B+n refers to n days after birth. D+n refers to n days after injection. **b** Histological workflow illustrating surgical procedure, whole-brain immunostaining, light-sheet imaging and quantification. M.p.i refers to months-post injection. **c** Workflow for electrophysiological assessment showing EEG recording, activity detection, and vigilance-controlled analysis readouts

To characterize and understand the neuropathological and neurophysiological effects of tau seeding in APP-KI mice, we established 2 cohorts of animals (Fig. 1): Histology (Fig. 1b) and Electrophysiology (Fig. 1c) cohorts. Each cohort consisted of 2 age groups (i.e., animals injected at 3-months-old or 6-months-old, n = 64 for electrophysiology and n = 48 for histology, for each age group), which were further comprised of 4 genotype-treatment groups (n = 16 for electrophysiology and n = 12 for histology): APP^NL-G-F/NL-G-F^ + tau seeding (NL-G-F-tau), APP^NL/NL^ + tau seeding (NL-tau), and respective buffer-injected controls (NL-G-F-Buffer and NL-Buffer). For the histology cohort, animals were sacrificed at 2 timepoints (1, or 5 months) after injection (Fig. 1a, n = 6 per age group per genotype-treatment group per timepoint). For the electrophysiology cohort, animals were recorded at the same time points as the histology readouts (i.e., 1 and 5 months after injection) and sacrificed at the end of the experiment. All cohorts and groups were equally sex-balanced. Animals were randomly assigned to each group using random number generation. To control for any confounds associated with the recording environment or order of recordings, at each recording timepoint, a recording schedule that randomized the recording cage, and order of the animals to be recorded, irrespective of sex, age, genotype or treatment, was generated using random number generation. Due to the exploratory pilot nature of this study, a reference study was not available for power analysis for a proper sample size estimation. The treatment conditions and genotypes of the animals were blinded to the experimenter until the point of data analysis.

### In vitro and ex vivo methods

#### AD-tau seed purification

Human brain samples were obtained from the University of Washington brain bank as a generous gift from Virginia M.Y. Lee and John Q. Trojanowski. The use of post-mortem brain tissues for research was approved by the University of Pennsylvania’s Institutional Review Board with informed consent from patients or their families. Purification methods for the preparation of AD-Tau seeds were followed according to the protocol listed in [25], and in accordance with relevant ethical guidelines. More details regarding patient profiles can be found in (Additional file 1: Table S1), as well as the protein quantification from each purification from patient samples (Additional file 1: Table S1). Tau seeding material was prepared from each patient sample on different days and verified for seeding capability by testing on mouse primary neurons. Additionally, western blotting and ponceau S staining was used to evaluate the seed purification protocol (Additional file 1: Fig. S3). The seeding material from 6 patients was pooled together and tested again for seeding on primary neurons before injecting into APP-KI animals. The same batch of seeding material was used throughout the entire study to control for batch-related differences.

#### Mouse brain clearing method

Mouse brain samples obtained following euthanasia were bisected along the midline and the hemisphere ipsilateral to the electrode implantation sites and/or injection site was placed into 15 ml flip-cap tubes. Fluorescent labelling and clearing of brain hemispheres were done based on the iDISCO+ protocol for all brains [26]. Hyperphosphorylated tau was specifically detected using an AT8 antibody (pSer202/Thr205/PSer208 [27], produced at Janssen Pharmaceutica) conjugated with a near-infrared fluorescent tag (PerkinElmer VivoTag 680XL) following the manufacturer’s protocol before labelling (9.18 µg/ml in 1.8 ml for 14 days per hemisphere). Pentameric formyl thiophene acetic acid (PFTAA) was used for selective staining of protein aggregates (30 µM in 1.8 ml for 1 day per hemisphere) [28]. Samples were stored in 5 ml tubes containing dibenzyl ether until imaging was carried out. An overview of the workflow can be seen in Fig. 1b.

#### Light-sheet microscopy

Cleared mouse brain samples were imaged using a light sheet microscope (Ultramicroscope II, Lavision Biotec GmbH), equipped with an Olympus MVPLAPO 2X (NA 0.50) objective lens and a DBE-corrected LV OM DCC20 dipping cap. Images were acquired with a Neo sCMOS camera (Andor) at a total magnification of 1.6X. Z-step were set at 10 µm, giving a voxel size of 4 µm^2^ × 10 µm. A linear blending algorithm was used to merge on the fly both left and right light sheets. Sagittal pictures were framed by 2-tiled mosaic was done using 488 nm, 561 nm and 640 nm emission lasers with 525/50 nm, 620/60 nm, and 680/30 nm emission filter respectively. The exposure time was defined and fixed at 100 ms and laser power was kept constant across batches.

#### Image analysis

The image semiquantitative analysis protocol used for the detection of both PFTAA and AT8 signals was adapted from ClearMap [29] and further refined from [30]. Subsequently, the total number of AT8-positive or PFTAA-positive voxels for a given brain region volume was calculated and expressed as a pathological load (% of voxels stained/total number of voxels in that region). Additionally, we dilated the binary segmented plaques by 5 pixels for defining the boundaries of plaque-associated AT8 versus non-plaque-associated AT8. AT8-positive tau within this boundary was considered plaque-associated tau. For every experiment, bar graphs expressing the pathological load (y-axis) were constructed with standard error of mean error bars. Pathology was evaluated in several brain regions of interest as delineated by mapping to the Mouse Allen Brain Atlas using Elastix [31, 32], namely: the hippocampal region, entorhinal area, isocortex and thalamus. For all graphs and analysis, the LabelRatio refers to the number of voxels stained divided by the total number of voxels in that entire brain region.

### In vivo methods

#### Surgical procedures

Surgeries were carried out when mice were 2–3 months old and 5–6 months old. Anaesthesia was induced via isoflurane inhalation (O2, N2O and 5% isoflurane), followed by the shaving of the fur and disinfection with Isobetadine (Meda Pharma SA, Belgium) and 70% Ethanol. Analgesics (dipidolor, 0.025 mg/kg, Xylocaine, 10%), and eye ointment (Opticorn A, EcuPhar BV, Belgium) were applied to the animal before insertion into a stereotactic frame. The animal was maintained under isoflurane during surgery (O2, N2O and 2–2.5% isoflurane), and kept at 37–38 °C using a heating pad. An incision was made on the skin along the sagittal plane to expose the skull, and sutures were used to hold the skin apart at the lateral edges of the opening. The tilt, yaw and roll of the head were adjusted by measuring the DV differential between the bregma and lambda sutures. All differentials were corrected to within 0.05 mm before drilling. Drilling locations were measured relative to bregma and drilled by hand. Stainless steel screws were affixed over the left frontal and right occipital lobes to secure the implant.

In order to determine if immediate effects of tau seeding were present in animals, there needed to be a way for obtaining an EEG recording baseline from the animals prior to seeding. This would involve the implantation of the EEG headstage, which would obstruct the injection of seeding material unless a cannula was also implanted at the same time the EEG headstage was implanted. And in order to render the histological and electrophysiological cohorts comparable to each other, both cohorts were implanted with the same cannula and injected in the same way.

For the histology cohort, the dura was punctured by a hypodermic needle (Precisionglide, 25 g, BD) followed by cannula implantation above the dorsal hippocampus (AP: − 2.2, ML: 1.8, DV: 1.7), (C315IA/SPC, 26G, Plastics One Inc.), fixed using dental cement (Relyx Unicem 2 cement, 3M United States) and cured with a dental light.

For the electrophysiology cohort, single polyamide-coated stainless-steel wire electrodes (100 μm diameter with a blunt tip, Peira bvba, Belgium) were implanted. The dura was punctured by a hypodermic needle (PrecisionGlide, 25 g, BD) at each location and implantation of electrodes were carried out with these coordinates: Medial entorhinal cortex (AP: − 4.8, ML: 3.25, DV: 2.2), Hippocampal CA1 (AP: − 2.2, ML: 1.8, DV: − 1.4), Retrosplenial cortex (AP: − 1.75, ML: 0.5, DV: 1.0), Thalamus (AP: − 0.83, ML: 0.75, DV: 2.75), and Reference (AP: − 1.0, ML: 1.2, DV: 0.8), followed by the cannula (AP: − 2.5, ML: 2.45, DV: 1.4, lateral angle of + 24.5 degrees,C315GAS-5/SPC, Plastics One Inc.). Each electrode was fixed with dental cement (Relyx Unicem 2 cement, 3M United States) and cured using a dental light. The ground screw electrode was implanted in the skull approximately 1 mm posterior to lambda. Subsequently, a multichannel connector (Nano strip connector, Omnetics, Minneapolis, USA) was connected to the electrodes and affixed using dental cement.

The skin surrounding the surgical site was sealed using veterinary glue (Vetbond, 3M United States) after either surgical procedure. Mice recovery was closely monitored until they were fully recovered (approximately ten to fourteen days).

#### Animal injections

Animals were injected with either AD-tau seeds or sterile phosphate-buffered saline (PBS) buffer solution via the cannula into the dorsal hippocampus at 3 or 6 months of age. Animals were anaesthetized and mounted into the stereotactic frame as in the surgical procedure. The needle used for injections was a Hamilton 10 μl syringe mounted onto an injection robot (StereoDrive, Neurostar, Germany). The injection needle was fitted with a custom tube and locking needle adaptor for the cannula (C315IAS-5/SPCm, Plastics One Inc.). Injection speed was set as 0.2 μl/min and a volume of 5 μl was injected, with a waiting time of 5 min after injection. Needles were tested for blockage by ejection of 0.1 μl of injectate before and after injection and changed once a blockage was noted. Animals were returned to home cages and monitored for 2 h after injection.

#### Animal euthanasia

Animals implanted with electrodes were electro-lesioned using a stimulator (STG4002-1.6 mA, MultiChannel Systems, GmbH) while under isoflurane anaesthesia (O2, N2O and 2–2.5% isoflurane). A detailed description of the electro-lesion settings can be found in Additional file 1: Methods M1. Animals were subsequently administered a dose of pentobarbital diluted in saline (120 mg/kg) and perfused with PBS mixed with heparin (10U/mL), followed by 4% paraformaldehyde (PFA). Brains were kept overnight in 4% PFA and washed two times in PBS for 15 min each before transferring to a solution of 0.1% sodium azide and stored at 4 °C.

#### Electrophysiological recording procedures

Mice were recorded in customized plexiglass chambers (modified from Med Associates Inc. Fairfax, Vermont). These Perspex boxes were placed in opaque sound-attenuated chambers fitted with a small ventilation fan and a house light. The entire home cage without the cover or food tray of the animal was placed above a plastic pedestal in the box. The house light was switched on during the entire recording duration. Each recording box was controlled by K-limbic software, (Med Associates, version 1.20.2). A video camera (uEye CP, IDS Imaging GmbH) was mounted on the top of the chamber to record animal behaviour.

Electrophysiological signals were acquired using a 4-channel wireless headstage (W2100-HS4, MultiChannel Systems GmbH) and interface board (W2100-IFB system, MultiChannel Systems GmbH) at a sampling rate of 1000 Hz. The wireless headstages were powered by a 30mAh battery (Wireless-B-30mAh, MultiChannel Systems GmbH). All signals recorded were referenced and grounded to the respective physical electrodes as described in the surgical procedures. Signals and battery levels (> 80%) were checked before recording. The MultiChannel Experimenter software (MultiChannel Systems GmbH, version 2.14.0.19346) was used to acquire the recordings and synchronize the video acquisition, which was carried out on a separate computer running the MultiChannel VideoControl software (MultiChannel Systems GmbH, version 2.2.0) at 25 Hz. The duration of each recording session was 1 h in length and carried out 2 h after the start of the dark phase. Animals were returned to the home rack after each session. Animals were recorded on 3 consecutive days at the same time each day to control for circadian effects and variability. Animals were recorded at 4 timepoints after surgical implantation: Pre-injection (4–6 h before injection), post-injection (1 day after injection), 1 month post-injection (30 days), and 5 months-post-injection (150 days).

#### Animal activity level estimation

To assess the activity state of the animal for subsequent analysis, the video files of each recording were processed to extract movement and activity information of the animal using DeepLabCut [33]. Once the model snapshot was finalized, the model was incorporated into a custom in-house software based on LabView (National Instruments, USA) for estimating the position of the animal and determining the activity level of the animal. A detailed protocol of the video pre-processing, tracking model, analysis, and activity level estimation can be found in Additional file 1: Methods M2.

The entire duration of each recording session was divided into 4 s epochs and classified as active or inactive based on whether the activity data crossed the threshold for a sufficient duration during each epoch. The classification of animal activity was a binary state of 0 (inactive) or 1 (active) with an activity threshold of 6.578 cm/s. The calculation of mouse velocity was calculated by taking the pixel difference between the centre of the tracking points between two consecutive frames multiplied by the frame rate of 25fps and pixel-distance scale factor (11.4 pixels per cm).

#### EEG data pre-processing and exclusion criteria

Local Field Potential (LFP) data were acquired from the electrophysiological recordings of the animal and divided into the same time-matched 4 s epochs as used for activity detection, and subsequently processed to remove noise and artefacts using MATLAB 2016a. Animals were also excluded based on noise and artefacts that could not be removed by processing. For a detailed description of the artefact and noise detection processing algorithms, as well as specific exclusion criteria, please refer to Additional file 1: Methods M3.

#### EEG analysis

EEG analysis was carried out on the LFP data with 3 primary endpoints: Power spectrum density estimation, phase-amplitude coupling and Higuchi fractal dimension analysis. Frequency bands definitions of delta (1–4 Hz), Theta-1 (4–6), Theta-2 (6–8) [34], Low Gamma (30–50 Hz) and High Gamma (51–80) [35] were used for the analysis of each readout. The information extracted from the LFP of each epoch was averaged across all epochs to generate an animal average.

Power spectrum density was estimated using the Welch method [36] with a Hamming window applied to the signal. The resulting power spectrum was median filtered across the power spectrum. The resulting power spectrum was subsequently log-normalized using a natural logarithm *ln* [37] to better meet the assumptions of a normal distribution for subsequent parametric statistical testing. The power density was estimated for each of the frequency bands listed above.

For phase-amplitude coupling, signals were convolved using complex Morlet wavelets to generate filtered signals in steps of 5 Hz from 10 to 200 Hz for the amplitude-modulated signals and steps of 0.5 Hz from 2 to 12 Hz for the phase modulating signals with a Morlet wavelet width of 7 cycles. Phase angles for each filtered signal were calculated using the angle() function in MATLAB. The modulation index (MI) was subsequently calculated based on the approach by [38] for each phase-amplitude signal pair to construct a matrix of MI values. The MI values were subsequently averaged across amplitude-frequency band pair ranges. The frequency band pair ranges used for averaging MI values were: Theta 1-Low Gamma, Theta 1-High Gamma, Theta 2-Low Gamma and Theta 2-High Gamma (as defined above).

Lastly, the calculation of the Higuchi fractal dimension (HFD) [39], a nonlinear approach to estimating the fractal complexity of a time series was carried out using a custom MATLAB script to calculate the HFD value for each 4-s epoch. The equation used for the estimation of the HFD is based on the original equation from [39], reviewed in [40]. The value of the free parameter kmax = 13 was derived based on the approach listed in [41].

#### Statistical analysis

Statistical analysis was performed in R v.4.0.5 using RStudio as the frontend for the development of analysis scripts.

To statistically evaluate if amyloid, tau (colocalized and non-colocalized) pathologies, as well as neurophysiological outcomes (i.e. power spectra, phase-amplitude coupling and Higuchi fractal dimension), were significantly influenced by the injection of AD-Tau seeds and other factors (e.g. Age at injection, Genotype, etc.), we fit a general linear mixed model (GLMM) to the respective dependent variable with Sex, Age at injection, Genotype, Treatment (seeded vs. buffer), Time post-injection, and Brain region as fixed effects (main effects), and the Age × Genotype × Treatment × Time post-injection × Brain region as interaction term (including all lower-order interactions that constitute this 5th-order interaction). More information regarding the statistical model can be found in the Statistical Analysis portion of the Methods section and Additional file 1: Methods M4 and M5.

To determine if the interaction effects were significant, a null model containing all other terms except for the interaction term of interest was compared to the model via log-likelihood. Significant interactions were determined if the null model was significantly different from the threshold (alpha = 0.05). This was repeated for all interaction terms of interest. Subsequent post-hoc pairwise comparisons were generated from the fitted model and adjusted using Benjamini–Hochberg False Discovery Rate (FDR) for multiple comparisons. The threshold for determination of significance was set at q = 0.05 for the multiple comparisons after correction using Benjamini–Hochberg FDR. All data were plotted using ggplot2 as bar graphs and dots representing individual animal values and error bars representing standard errors.

## Results

### APP-KI^NL-G-F^ animals develop AT8-positive tau pathology longitudinally over 5 months following injection of tau seeds

We first aimed to characterize the spatiotemporal development of both tau and amyloid pathology. In this regard, we established a cohort of APP-KI^NL-G-F^ and APP-KI^NL-NL^ animals of 2 different age groups: 3- and 6-months of age (refer to Methods for specific animal cohorts and numbers), and injected them with either AD-tau seeding material or buffer solution (i.e., phosphate-buffered saline) into the hippocampus, resulting in a total of 4 genotype-treatment animal groups: NL-G-F animals injected with tau seeds (NL-G-F-tau), NL-G-F animals injected with buffer solution (NL-G-F-buffer), NL/NL animals seeded with tau (NL-tau) and NL/NL animals injected with buffer solution (NL-buffer). Animals were sacrificed at 1 and 5 months post injection (m.p.i) to evaluate the presence and quantity of both amyloid and tau pathology in the hippocampus, entorhinal cortex, isocortex, and thalamus using light sheet microscopy.

We assessed tau pathology through the usage of an AT8 antibody, a marker for phosphorylated tau at the Ser 202 and Thr 205 residues [42] as a measure for tau pathology and amyloid pathology, Pentameric formyl thiophene acetic acid (PFTAA) [43]. We measured the regional amount of AT8 positive tau and amyloid pathology (measured as the number of voxels stained positive for AT8 or PFTAA divided by the total number of voxels in that region, see Methods for more details).

We examined which factors may contribute to the development of pathology by testing if significant interactions were present using a GLMM. The amount of amyloid or AT8-positive tau pathology was fit with Age at injection (referred to as Age in the model), Genotype, Treatment (tau seeding or buffer injection), Time post-injection and Brain region as fixed effects and the Age × Genotype × Treatment × Time post-injection × Brain region as interaction term (including all lower-order interactions that constitute this 5th-order interaction) (see Methods and Additional file 1: Methods for more information).

The 5-way interaction of Age × Genotype × Treatment × Time post-injection × Brain region interaction term was not significant for AT8-positive tau pathology (using alpha = 0.05), (i.e., ${\chi }^{2}\left(3\right)=$ 1.302105, *p* = 0.7286). After excluding the highest order interaction term and refitting the model, the significance of the lower-order interactions terms was tested. The 4-way interaction between Brain Region × Genotype × Treatment × Time post-injection interaction was statistically significant (${\chi }^{2}\left(3\right)=44.00339,$ *p* value < 0.0001), but not other 4-way interactions. This interaction suggests that AT8-positive tau pathology develops differently across time, unequally between APP-KI^NL-G-F^ and APP-KI^NL-NL^ animals and does not develop at the same rate across different brain regions, and was not significantly influenced by the age at which the animal was injected.

When examining which factors contribute to the development of amyloid pathology, the 5-way interaction between Age × Genotype × Treatment × Time post-injection × Brain region was significant (${\chi }^{2}\left(3\right)=18.26804,$ *p* value = 0.00038). Interestingly, this interaction effect was significant and suggested that the injection of AD-tau influences the development of amyloid plaque pathology, while also being influenced by the age at which the animal was injected, and brain region, and also develops differently across time. The results of these interactions are examined in more detail in the subsequent sections.

Subsequently, we also sought to understand if the initial amount of amyloid plaque pathology, which correlates proportionately with the age of the animal, would affect the subsequent development of both plaque-associated and non-associated tau pathology. We report that there were no significant interactions terms containing age as a factor in the GLMM for assessing tau pathology, suggesting that age does not significantly affect the amount of tau pathology. In addition, the main effect of Age was non-significant (i.e., *p* = 0.3641).

**Fig. 2:**
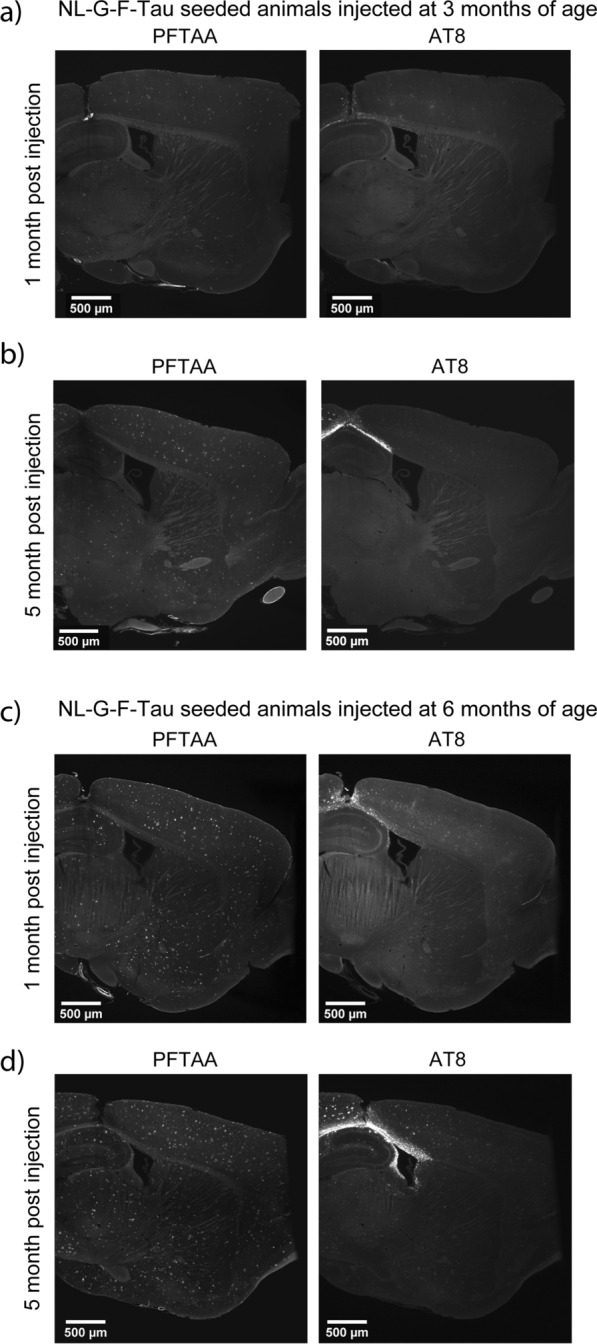
Representative histological images from NL-G-F-Tau animals injected at 3 and 6 months of age. Representative light sheet images of amyloid (PFTAA) and AT8-positive tau pathology in 3-month-old NL-G-F-tau animals at **a** 1 month-post-injection and **b** 5 months-post-injection. Representative light sheet images of amyloid and AT8-positive tau pathology in 6-month-old NL-G-F-tau animals at **c** 1 month-post-injection and **d** 5 months-post-injection. PFTAA refers to Pentameric formyl thiophene acetic acid, AT8 refers to AT8-positive tau pathology

The injection of AD-tau seeding resulted in the progressive development of AT8-positive pathology in NL-G-F-tau animals injected at 3 months of age, from 1 to 5 m.p.i (Fig. 2a, b) and in NL-G-F-tau animals injected at 6 months of age from 1 to 5 m.p.i (Fig. 2c, d) in the isocortex and entorhinal area. Notably, the amount of AT8-positive tau pathology was not significantly different in the hippocampus of NL-G-F-tau mice between 1 and 5 m.p.i in either age group. Representative images of NL-Tau, NL-Buffer, NL-G-F-buffer from animals injected at 3 and 6 months of age, at 1 and 5 m.p.i can be found in Additional file 1: Fig. S1, and higher magnification images can be found in Additional file 1: Fig. S4.

**Fig. 3:**
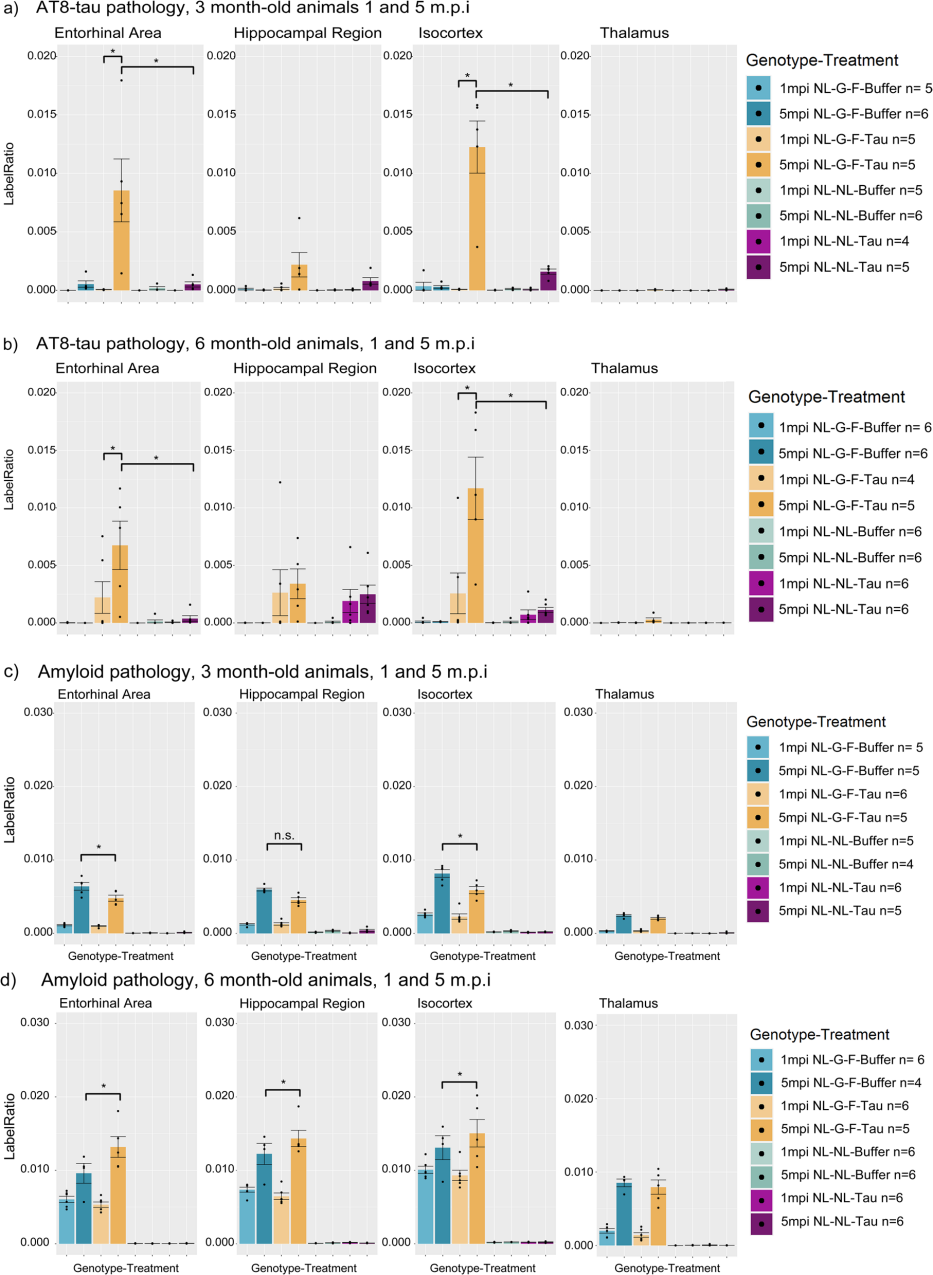
Quantification and comparisons of AT8 and amyloid pathology across genotype, and time-post-injection from animals injected at 3 or 6 months of age. **a** Quantification and pairwise comparisons of AT8-positive tau pathology in animals injected at 3 months of age, at 1 and 5 m.p.i. **b** Quantification and pairwise comparisons of AT8-positive tau pathology in in animals injected at 6 months of age, at 1 and 5 m.p.i. **c** Quantification and pairwise comparisons of amyloid pathology in animals injected at 3 months of age, at 1 and 5 m.p.i. **d** Quantification and pairwise comparisons of amyloid pathology in animals injected at 6 months of age, at 1 and 5 m.p.i. Error bars are standard error of mean. Asterisks indicate significant comparisons (*p* < 0.05). n.s. refers to non-significant comparisons. M.p.i refers to months-post injection

A quantification and subsequent pairwise comparison between the 1 and 5 m.p.i timepoints revealed that NL-G-F animals exhibited significantly more AT8 pathology in the Isocortex and Entorhinal areas, but not in the Hippocampus in animals injected at either 3 or 6 months of age (Fig. 3a, b, Table 1a).

**Table 1:** Table containing pairwise comparisons of quantified amyloid and AT8-positive tau pathology: (a) comparison of AT8-positive tau pathology in NL-G-F-Tau and NL-Tau mice between 1 and 5 m.p.i. across brain regions, (b) comparison of AT8-positive tau pathology between NL-G-F-Tau and NL-Tau animals at 5 m.p.i, (c) comparison of amyloid pathology between NL-G-F-tau and NL-G-F-buffer animals at 5 m.p.i

Brain region	Pairwise comparison	Estimate	SE	df	T ratio	*P* value
*(a) Post hoc pairwise contrasts of AT8-positive tau pathology in NL-G-F and NL-NL animals between 1 and 5 m.p.i*						
Entorhinal area	3mo NL-G-F-Tau 1mpi–3mo NL-G-F-Tau 5mpi	− 0.0078	0.0009	69	− 8.2517	1.0193E−10
	6mo NL-G-F-Tau 1mpi–6mo NL-G-F-Tau 5mpi	− 0.0053	0.0009	69	− 5.5507	4.2413E−06
Isocortex	3mo NL-G-F-Tau 1mpi–3mo NL-G-F-Tau 5mpi	− 0.0119	0.0009	69	− 12.5660	3.2351E−17
	6mo NL-G-F-Tau 1mpi–6mo NL-G-F-Tau 5mpi	− 0.0095	0.0009	69	− 10.0039	8.1906E−14
Hippocampal region	No significant differences					
Thalamus	No significant differences					
*(b) Post hoc pairwise contrasts of AT8-positive tau pathology in NL-G-F animals between 3 and 6 months of age at 5 m.p.i*						
Entorhinal area	3mo NL-G-F-Tau 5mpi–3mo NL-Tau 5mpi	0.0075	0.0010	69	7.5953	1.3175E−09
	6mo NL-G-F-Tau 5mpi–6mo NL-Tau 5mpi	0.0069	0.0009	69	7.3084	4.0347E−09
Isocortex	3mo NL-G-F-Tau 5mpi–3mo NL-Tau 5mpi	0.0107	0.0010	69	10.9388	2.0638E−15
	6mo NL-G-F-Tau 5mpi–6mo NL-Tau 5mpi	0.0105	0.0009	69	11.1057	1.1349E−15
Hippocampal formation	No significant differences					
Thalamus	No significant differences					
*(c) Post hoc pairwise contrasts of amyloid pathology between NL-G-F-tau and NL-G-F-buffer animals at 5 m.p.i*						
Entorhinal area	3mo NL-G-F-Buffer 5mpi–3mo NL-G-F-Tau 5mpi	0.0016	0.0007	69	2.3416	3.6999E−02
	6mo NL-G-F-Buffer 5mpi–6mo NL-G-F-Tau 5mpi	− 0.0036	0.0007	69	− 5.1335	5.2315E−06
Isocortex	3mo NL-G-F-Buffer 5mpi–3mo NL-G-F-Tau 5mpi	0.0022	0.0007	69	3.3480	2.4457E−03
	6mo NL-G-F-Buffer 5mpi–6mo NL-G-F-Tau 5mpi	− 0.0020	0.0007	69	− 2.8146	1.0855E−02
Hippocampal formation	6mo NL-G-F-Buffer 5mpi–6mo NL-G-F-Tau 5mpi	− 0.0021	0.0007	69	− 3.0085	6.4259E−03
Thalamus	No significant differences					

SE refers to standard error. Df refers to degrees of freedom. Estimate refers to the estimated difference in value between pairwise comparisons. mo refers to months-old at injection

As expected, older APP-KI^NL-G-F^ mice exhibited more amyloid pathology than younger mice, as well as a lack of amyloid pathology in APP-KI^NL-NL^ mice at the timepoints evaluated (Fig. 3c, d, Additional file 1: Fig. S1).

In NL-Tau animals, the amount of AT8-positive tau pathology was not significantly different between 1 and 5 m.p.i in either age group in all regions quantified, suggesting that amyloid plaque pathology likely facilitates the development of AT8-positive tau pathology. However, there also exists the possibility that the presence of pathogenic amyloid beta oligomers could also influence the development of AT8-positive tau. Given the correlation between the amount of plaque pathology, and oligomeric amyloid beta, disentangling the contribution of oligomeric species is tricky.

### NL-G-F-Tau animals exhibit increased AT8-positive tau pathology compared to NL-Tau animals after tau seeding

Significantly more AT8-positive tau pathology was noted in amyloid plaque-bearing mice compared to non-amyloid bearing mice (i.e., more AT8-positive tau pathology in NL-G-F-tau mice compared to NL-tau mice) even when accounting for the same amount of time following injection. This is illustrated when comparing NL-G-F-tau and NL-tau animals, at the same age and time post-injection (Fig. 3a, b, Table 1b), notably at the 5 m.p.i timepoints. The hippocampus and thalamus did not show similarly significant differences. These results indicate that the genotype, which correlates with the presence of amyloid pathology facilitates the presence of AT8-tau pathology, with regional specificity.

### Amyloid pathology is influenced by the injection of AD-tau seeds in NL-G-F-Tau animals and is also differentially influenced by age

Interestingly, the interaction effect of Age × Genotype × Treatment × Time post-injection × Brain region on amyloid pathology indicates an effect of tau seeding on amyloid pathology, which is also influenced by the brain region.

We report that NL-G-F-Tau animals have significantly different levels of amyloid pathology at 5 m.p.i compared to buffer-injected conditions. This relationship was not present at 1 m.p.i, suggesting it to be a longitudinal effect of the tau seeding (Fig. 3c, d, Table 1c). Animals injected at 3-months of age exhibited significantly reduced amyloid plaque pathology in the entorhinal area and isocortex (Fig. 3c, Table 1c) whereas animals injected at 6 months of age exhibited significantly increased amyloid pathology in the entorhinal area, isocortex and hippocampus (Fig. 3d, Table 1c). Additionally, this relationship appeared to be reinforced by the lack of a significant difference in the thalamus, which does not exhibit significant AT8-positive tau pathology at any timepoints nor any significant differences in amyloid pathology.

In the previous section, we noted that there were no significant differences in the levels of AT8-positive tau pathology between animals injected at 3 and 6 months of age. However, this does not distinguish between 2 forms of AT8-positive tau: plaque-associated tau and non-associated tau [25], which could be related to changes in amyloid pathology. Thus, we quantified and statistically assessed the amount of plaque-associated and non-plaque-associated tau to determine if there were differences that could explain this age-dependent effect. Neither the amount of plaque-associated tau (Additional file 1: Fig. S2a, Table S2a) nor non-plaque-associated tau (Additional file 1: Fig. S2b, Table S2b) were significantly different between animals injected at 3 and 6 months of age at 5 m.p.i.

These results suggest that while the seeding appears to affect the amount of amyloid pathology, the age-dependent effect of tau seeding on amyloid pathology does not appear to be directly explained by the amount of plaque-associated or non-associated AT8-positive tau pathology. Thus, we sought to understand if neurophysiological readouts could provide a possible insight into this relationship.

### Tau seeding significantly alters neurophysiological outcomes that are influenced by the age at which the animal was injected and the time after injection

Subsequently, we sought to understand if these pathological changes translated to neurophysiological consequences and if any neurophysiological readouts could provide insight into why older NL-G-F-tau animals eventually develop more amyloid pathology. To this end, we established another cohort of identically aged APP-KI animals, with the same genotypes, seeding conditions, and age groups as the previously characterized histology cohort. We subsequently implanted electrodes into several brain regions corresponding to the hippocampus, medial entorhinal cortex, retrosplenial cortex and thalamus (see Methods for specific coordinates of regions). We subsequently recorded LFPs across several timepoints: Pre-injection (1 day before injection), post-injection (1 day after injection), and 1, and 5-months after injection. Recordings were subsequently processed and analyzed for several readouts: EEG power spectra density, phase-amplitude coupling, and HFD scores. Subsequently, to test which factors may be contributing to changes associated with neurophysiological outcomes, we fit a GLMM to these readouts in the same manner as the histological characterization to determine if significant interaction effects were present.

For each sub-readout, a 5-way interaction between Age × Genotype × Treatment × Time post injection × Brain region (including all lower-order interactions that constitute this 5th-order interaction) was investigated. A 5-way interaction for low gamma power was noted to be significant (${\chi }^{2}\left(9\right)=17.78767$, *p* value = 0.0377). For other readouts that did not contain significant 5-way interactions, the highest order non-significant interaction term was excluded, and the model refitted, followed by testing the significance of the lower-order interactions terms in a hierarchical stepwise way.

For readouts of power spectra density, significant 4-way interactions between Age × Electrode × Genotype × Treatment were present for the readouts of Theta 1 (${\chi }^{2}\left(3\right)=14.89794,$ p value = 0.0019), Theta 2 (${\chi }^{2}\left(3\right)=13.91431,$ *p* value = 0.003), Delta (${\chi }^{2}\left(3\right)=11.35379,$ *p* value = 0.01) and High Gamma (${\chi }^{2}\left(3\right)=7.792645,$ *p* value = 0.05) power values.

Other significant 4-way interactions between Age × Genotype × Treatment × Time post-injection for the measures of Low Gamma (${\chi }^{2}\left(4\right)=17.01485,$ *p* value = 0.0019), and High Gamma power (${\chi }^{2}\left(4\right)=13.65165,$ *p* value = 0.0085), were noted.

For the readouts of phase-amplitude coupling, of the 4th-order interactions tested, only a significant interaction of Age × Genotype × Electrode × Treatment was noted for the readout of Theta2-High Gamma coupling (i.e., ${\chi }^{2}\left(3\right)= 26.72049, p\mathrm{ value}<0.0001$) was noted.

For the readout of Higuchi Fractal Dimension (HFD), no 4-way interactions were noted to be significant, suggesting a lack of an interaction effect of interest.

Notably, of these readouts tested, power spectra changes appear to be associated with an interaction effect of treatment, age, genotype, as well time-post-injection, suggesting a longitudinal effect associated with tau seeding. The interaction effects are examined in more detail in the following sections.

### APP^NL-G-F^ animals exhibit band-specific baseline power spectra deficiencies at older ages compared to APPNL^/NL^ animals.

**Fig. 4:**
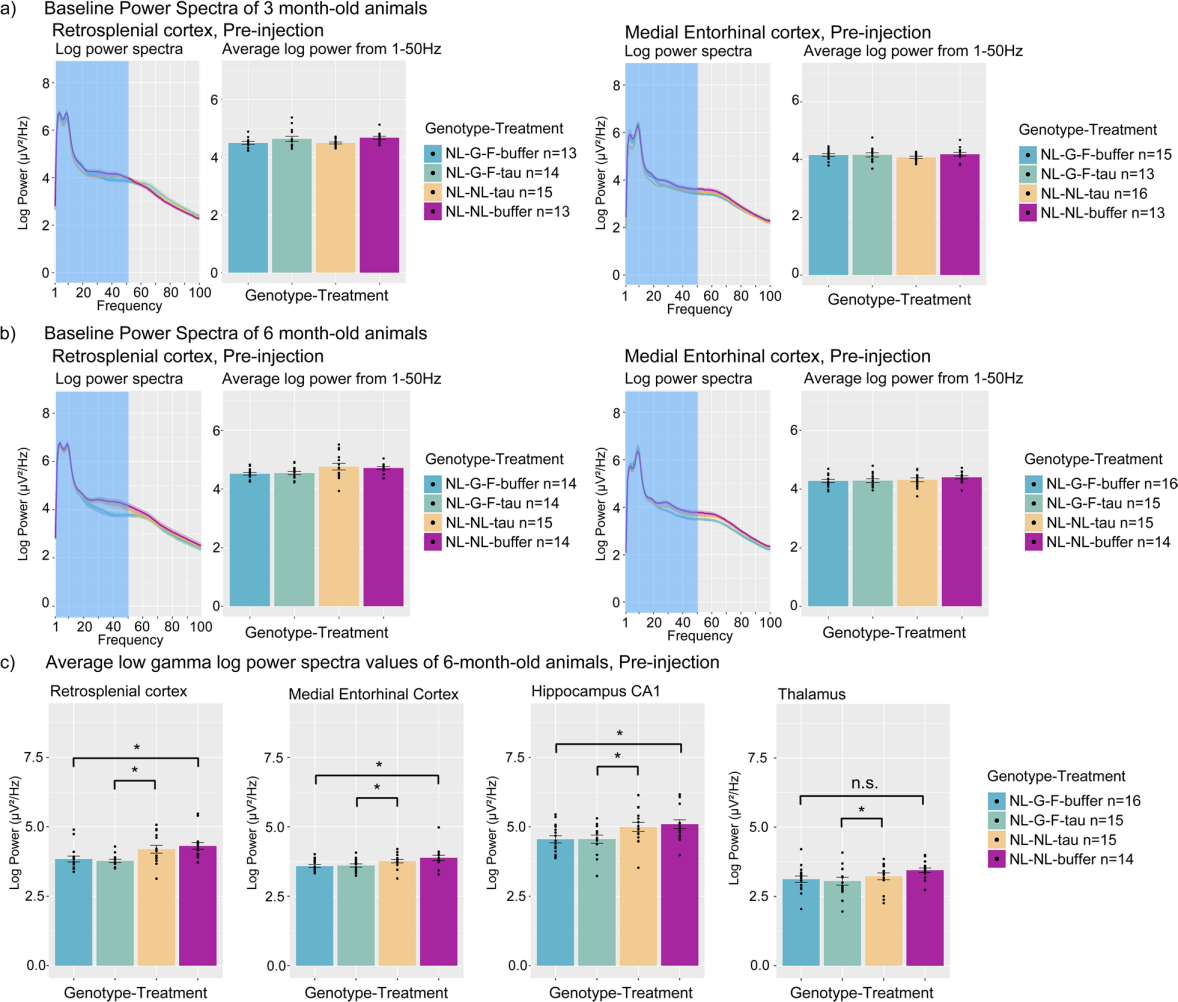
Power spectra quantification and comparisons between NL-G-F and NL-NL animals at baseline. Log power graphs and power spectra comparisons of NL-G-F and NL-NL animals from 1 to 50 Hz injected at **a** 3 months of age and **b** 6 months of age from the medial entorhinal cortex and retrosplenial cortex before injection. Bar plot values correspond to quantified average of blue highlighted regions. **c** Baseline low gamma power spectra values before tau seeding injection in 6-month-old APP-KI animals, across multiple brain regions. Error bars are standard error of mean. Asterisks indicate significant comparisons (*p* < 0.05)

We evaluated power spectra between plaque-positive APP-KI^NL-G-F^ and plaque-absent APP-KI^NL-NL^ mice before injection to determine if any baseline differences were present. At pre-injection recordings, we report that both APP-KI^NL-G-F^ and APP-KI^NL-NL^ mice did not show statistically significant differences when comparing power spectra from 1 to 50 Hz in either age group (Fig. 4a, b, Table 2a) in either the retrosplenial cortex or medial entorhinal cortex nor in the hippocampus or thalamus (Table 2a) suggesting no overt power spectra differences.

**Table 2:** Table of pairwise comparisons of baseline power spectra values from 1 to 50 Hz and low gamma frequency band range: (a) pairwise comparisons of baseline power spectra values from 1 to 50 Hz in between NL-G-F and NL-NL animals indicating no significant differences, (b) pairwise comparisons of baseline low gamma power spectra values in animals to be seeded at 6 months of age, NL-G-F and NL-NL animals

Electrode location	Pairwise comparison	Estimate	SE	df	T ratio	*P* value
*(a) Post hoc pairwise contrasts of 1–50 Hz power spectra values at baseline*						
Entorhinal area	3mo NL-G-F-Buffer Pre–3mo NL-NL-Buffer Pre	0.3187	0.1818	116	1.7527	1.2681E−01
	3mo NL-G-F-Tau Pre–3mo NL-NL-Tau Pre	− 0.1970	0.1870	116	− 1.0534	3.8812E−01
	6mo NL-G-F-Buffer Pre–6mo NL-NL-Buffer Pre	0.2375	0.1828	116	1.2996	2.7450E−01
	6mo NL-G-F-Tau Pre–6mo NL-NL-Tau Pre	0.1870	0.1828	116	1.0230	4.0373E−01
Retrosplenial cortex	3mo NL-G-F-Buffer Pre–3mo NL-NL-Buffer Pre	0.0973	0.1818	116	0.5352	6.8099E−01
	3mo NL-G-F-Tau Pre–3mo NL-NL-Tau Pre	− 0.1678	0.1892	116	− 0.8869	4.7571E−01
	6mo NL-G-F-Buffer Pre–6mo NL-NL-Buffer Pre	0.0983	0.1828	116	0.5379	6.7922E−01
	6mo NL-G-F-Tau Pre–6mo NL-NL-Tau Pre	− 0.0460	0.1850	116	− 0.2487	8.5523E−01
Hippocampal formation	3mo NL-G-F-Buffer Pre–3mo NL-NL-Buffer Pre	0.0987	0.1818	116	0.5427	6.7634E−01
	3mo NL-G-F-Tau Pre–3mo NL-NL-Tau Pre	0.2752	0.1870	116	1.4714	2.0967E−01
	6mo NL-G-F-Buffer Pre–6mo NL-NL-Buffer Pre	0.1285	0.1828	116	0.7032	5.8077E−01
	6mo NL-G-F-Tau Pre–6mo NL-NL-Tau Pre	0.0568	0.1850	116	0.3072	8.2146E−01
Thalamus	3mo NL-G-F-Buffer Pre–3mo NL-NL-Buffer Pre	0.2593	0.1818	116	1.4259	2.2588E−01
	3mo NL-G-F-Tau Pre–3mo NL-NL-Tau Pre	0.1300	0.1879	116	0.6922	5.8712E−01
	6mo NL-G-F-Buffer Pre–6mo NL-NL-Buffer Pre	0.3864	0.1828	116	2.1145	6.0009E−02
	6mo NL-G-F-Tau Pre–6mo NL-NL-Tau Pre	− 0.0003	0.1828	116	− 0.0017	9.9916E−01
*(b) Post hoc pairwise contrasts of low gamma power spectra values at baseline*						
Entorhinal area	6mo NL-G-F-Buffer Pre–6mo NL-NL-Buffer Pre	− 0.2466	0.1116	118	− 2.2094	3.8786E−02
	6mo NL-G-F-Tau Pre–6mo NL-NL-Tau Pre	− 0.3409	0.1120	118	− 3.0429	4.3913E−03
Retrosplenial cortex	6mo NL-G-F-Buffer Pre–6mo NL-NL-Tau Pre	− 0.3358	0.1266	118	− 2.6521	1.3000E−02
	6mo NL-G-F-Tau Pre–6mo NL-NL-Tau Pre	− 0.4319	0.1122	118	− 3.8495	3.3214E−04
Hippocampal formation	6mo NL-G-F-Buffer Pre–6mo NL-NL-Buffer Pre	− 0.4677	0.1123	118	− 4.1661	1.0783E−04
	6mo NL-G-F-Tau Pre–6mo NL-NL-Tau Pre	− 0.4748	0.1129	118	− 4.2070	9.2651E−05
Thalamus	6mo NL-G-F-Buffer Pre–6mo NL-Buffer Pre	− 0.1635	0.1116	118	− 1.4648	0.1747
	6mo NL-G-F-Tau Pre–6mo NL-Tau Pre	− 0.4273	0.1121	118	− 3.8130	0.0004

SE refers to standard error. Df refers to degrees of freedom. Estimate refers to the estimated difference in value between pairwise comparisons. Pre refers to pre-injection recordings

Subsequently, we investigated individual power bands of delta, theta, and gamma (see Methods for frequency band ranges) to determine if band-specific changes were present. No significant differences in any power band were noted for all comparisons in animals injected at 3 months of age before injection. However, in animals to be injected at 6 months of age, significant differences in the low gamma power were present when comparing APP-KI^NL-G-F^ and APP-KI^NL-NL^ animals at baseline. This was localized to both the retrosplenial cortex, hippocampus and medial entorhinal cortex and thalamus of these animals (Fig. 4c, Table 2b), except for the NL-G-F-Buffer and NL-Buffer comparisons, indicating that gamma power deficits are present in the retrosplenial cortex, hippocampus and medial entorhinal cortex of older APP-KI^NL-G-F^ animals.

### AD-tau seeding injections result in immediate impairments of power spectra and phase-amplitude coupling in the hippocampus of mice

Next, we sought to understand the immediate effects of tau seeding injections in APP-KI animals by comparing neurophysiological readouts before injection and 1 day following injection. We compared power spectra, HFD scores and phase-amplitude coupling results before injection and one day after injection within the same genotype-treatment groups to determine if alterations in these readouts were present immediately following injection.

**Fig. 5:**
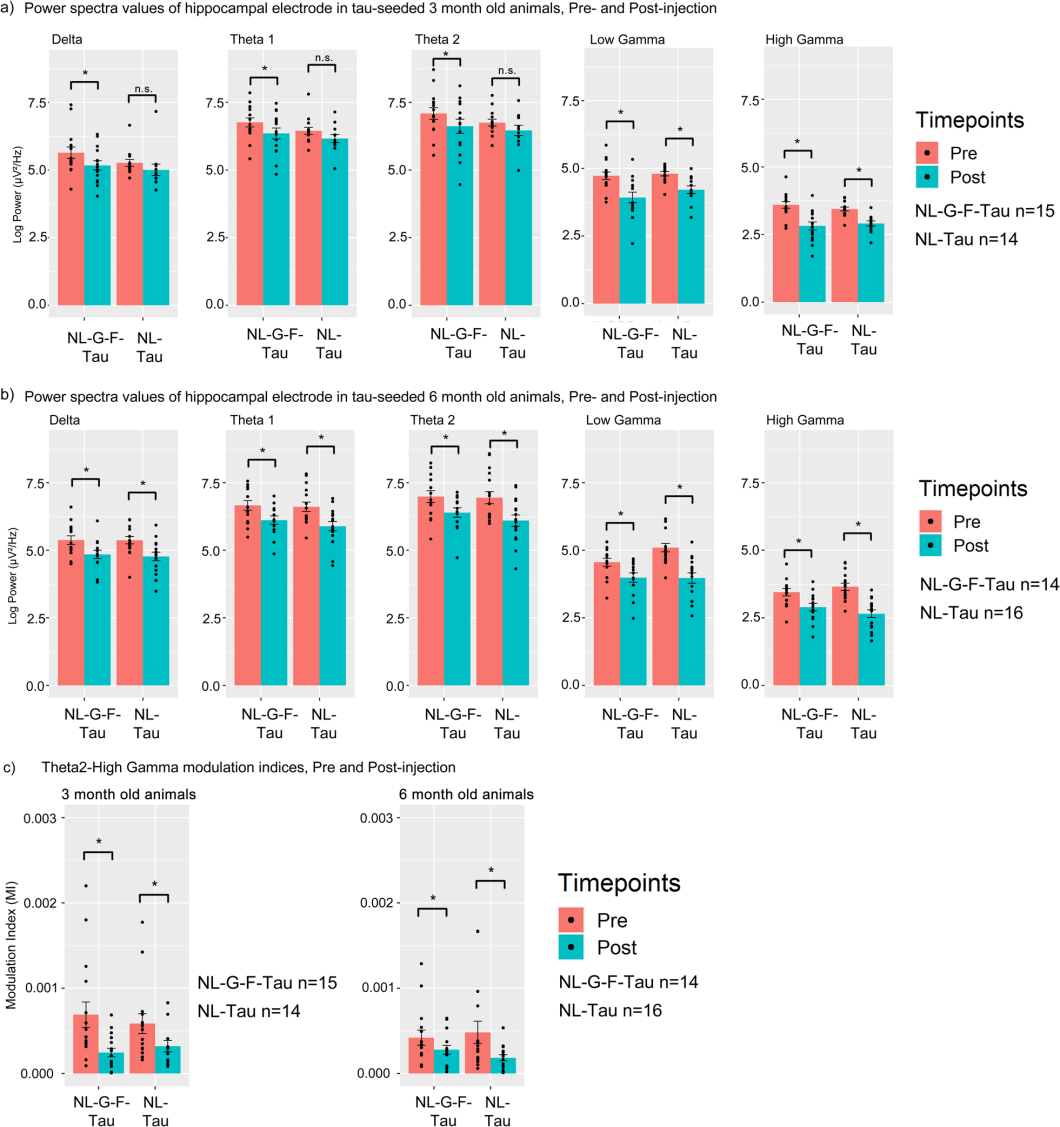
Bar plots of power spectra changes and phase-amplitude coupling and in 3- and 6-month-old APP-KI animals before and 1 day after injection. Bar plots of hippocampal power values across different frequency bands in animals injected at **a** 3- and **b** 6-months of age before and 1 day after injection. c) Phase-amplitude coupling deficits immediately following injection in tau-seeded APP-KI animals at either 3 or 6 months of age. Error bars are standard error of mean. Asterisks indicate significant comparisons (*p* < 0.05). Pre refers to pre-injection. Post refers to 1 day after injection

Except for NL-tau animals injected at 3 months of age, AD-tau seed-injected animals exhibited significantly decreased power in Delta, Theta-1, Theta-2, low and high gamma power bands in animals injected at 3 and 6 months of age, 1 day immediately after injection (Fig. 5a, b, Table 3a) in the hippocampus. In NL-tau animals injected at 3 months of age, only low and high gamma power was consistently significantly reduced in the hippocampus. Significant differences in Delta, Theta-1, and Theta-2 power were not noted in NL-Tau animals injected at 3 months of age. Reductions in power were not noted in buffer-injected animals, nor in non-hippocampal regions, indicating a localized effect associated with the injection of tau seeds.

**Table 3:** Table of pairwise comparisons of power spectra values before and 1 day immediately after injection: (a) pairwise comparisons of power spectra values of delta, theta and gamma power values before and after injection, (b) pairwise comparisons of phase-amplitude coupling of Theta 2-High Gamma modulation indices before and after injection

Frequency band	Pairwise comparison	Estimate	SE	df	T ratio	*P* value
*(a) Post hoc pairwie contrasts of hippocampal power spectra values, pre and post injection*						
Delta	3mo NL-G-F-Tau Post–3mo NL-G-F-Tau Pre	− 0.4741	0.1503	784	− 3.1544	3.4965E−03
	6mo NL-G-F-Tau Post–6mo NL-G-F-Tau Pre	− 0.5309	0.1556	784	− 3.4128	1.5923E−03
	3mo NL-NL-Tau Post–3mo NL-NL-Tau Pre	− 0.4224	0.1640	784	− 2.5759	1.8642E−02
	6mo NL-NL-Tau Post–6mo NL-NL-Tau Pre	− 0.6009	0.1455	784	− 4.1293	1.2635E−04
Theta 1	3mo NL-G-F-Tau Post–3mo NL-G-F-Tau Pre	− 0.4105	0.1366	784	− 3.0043	5.2680E−03
	6mo NL-G-F-Tau Post–6mo NL-G-F-Tau Pre	− 0.5427	0.1414	784	− 3.8369	2.9373E−04
	3mo NL-NL-Tau Post–3mo NL-NL-Tau Pre	− 0.4037	0.1491	784	− 2.7070	1.2703E−02
	6mo NL-NL-Tau Post–6mo NL-NL-Tau Pre	− 0.7166	0.1323	784	− 5.4157	3.0870E−07
Theta 2	3mo NL-G-F-Tau Post–3mo NL-G-F-Tau Pre	− 0.4686	0.1565	784	− 2.9942	6.1932E−03
	6mo NL-G-F-Tau Post–6mo NL-G-F-Tau Pre	− 0.5905	0.1620	784	− 3.6450	8.0361E−04
	3mo NL-NL-Tau Post–3mo NL-NL-Tau Pre	− 0.3546	0.1707	784	− 2.0771	6.5286E−02
	6mo NL-NL-Tau Post–6mo NL-NL-Tau Pre	− 0.8470	0.1515	784	− 5.5896	2.9056E−07
Low Gamma	3mo NL-G-F-Tau Post–3mo NL-G-F-Tau Pre	− 0.7983	0.1343	784	− 5.9449	1.9928E−08
	6mo NL-G-F-Tau Post–6mo NL-G-F-Tau Pre	− 0.5682	0.1390	784	− 4.0874	1.2294E−04
	3mo NL-NL-Tau Post–3mo NL-NL-Tau Pre	− 0.6172	0.1463	784	− 4.2191	7.2208E−05
	6mo NL-NL-Tau Post–6mo NL-NL-Tau Pre	− 1.1232	0.1300	784	− 8.6379	6.2262E−16
High Gamma	3mo NL-G-F-Tau Post–3mo NL-G-F-Tau Pre	− 0.7792	0.1133	784	− 6.8783	1.8638E−10
	6mo NL-G-F-Tau Post–6mo NL-G-F-Tau Pre	− 0.5535	0.1173	784	− 4.7206	1.1868E−05
	3mo NL-NL-Tau Post–3mo NL-NL-Tau Pre	− 0.5450	0.1236	784	− 4.4108	4.2871E−05
	6mo NL-NL-Tau Post–6mo NL-NL-Tau Pre	− 0.9976	0.1097	784	− 9.0956	9.0178E−17
*(b) Post hoc pairwise contrasts of Theta2-High gamma coupling in hippocampus of tau-seeded mice, pre- and post-injection*						
Hippocampus	3mo NL-G-F-Tau Post–3mo NL-G-F-Tau Pre	− 0.0003	0.0001	3492	− 3.6237	1.4275E−03
	6mo NL-G-F-Tau Post–6mo NL-G-F-Tau Pre	− 0.0003	0.0001	3492	− 3.1300	6.5037E−03
	3mo NL-NL-Tau Post–3mo NL-NL-Tau Pre	− 0.0003	0.0001	3492	− 3.2444	4.6307E−03
	6mo NL-NL-Tau Post–6mo NL-NL-Tau Pre	− 0.0003	0.0001	3492	− 2.9325	1.1000E−02

Pre refers to pre-injection values, post refers to post-injection values. SE refers to standard error. Df refers to degrees of freedom. Estimate refers to the estimated difference in value between pairwise comparisons

Additionally, phase-amplitude coupling changes were also noted immediately following the injection of AD-tau seeds. Significant reductions in modulation indices were noted to be present in Theta2-High Gamma coupling measures, in both age groups (Fig. 5c, Table 3b). These results indicate that the injection of seeding material already causes immediate impairments of hippocampal function in terms of phase-amplitude coupling and power spectra changes.

### AD-Tau injected animals injected at a younger age exhibit lasting hippocampal impairment in power spectra, and phase-amplitude coupling deficits appear at later time points after injection

We sought to understand the longitudinal neurophysiological effects associated with tau seeding, amyloid, and tau pathology by recording the local field potentials (LFPs) of these animals at 1-and 5-month post-injection, corresponding to the time points when histology was evaluated, to determine if changes in neurophysiology could be associated with the presence and amount of pathology. The interaction effect of Age × Genotype × Treatment × Time post-injection on low and high gamma power suggested that changes in low gamma and high gamma power appear to be differentially influenced by the age at which the seeding took place, as well as the duration after injection.

**Fig. 6:**
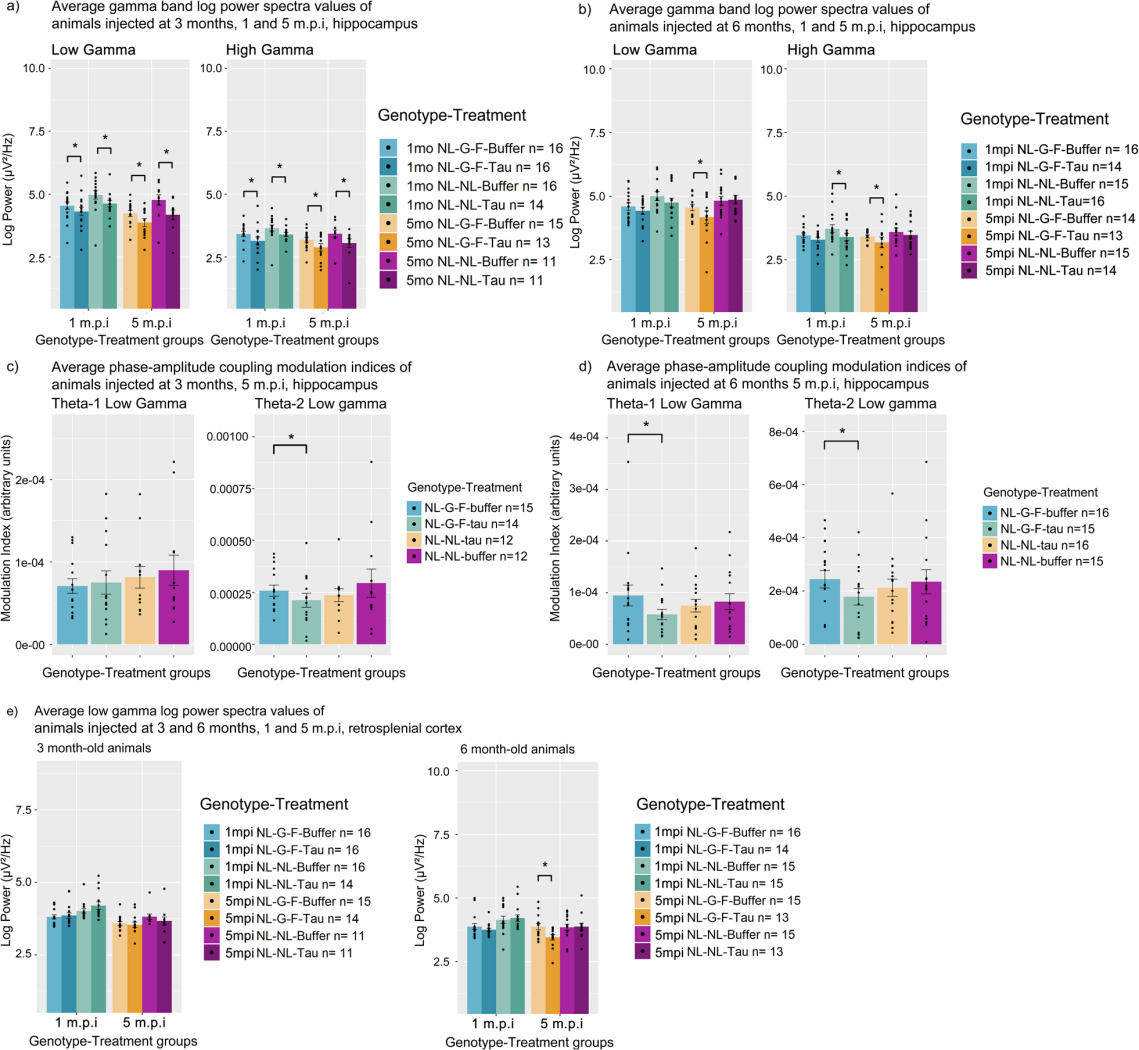
Bar plots of longitudinal changes in power spectra and phase-amplitude coupling at 1 and 5 months-post injection. **a** Bar plots of hippocampal low and high gamma band power of animals seeded at 3 months of age at 1 and 5 months-post-injection. **b** Bar plots of hippocampal low and high gamma band power of animals seeded at 6 months of age at 1 and 5 months-post-injection. Theta1-low gamma and Theta-2 low gamma phase-amplitude coupling comparisons in the hippocampus **c** of animals seeded at 3 months of age and **d** of animals seeded at 6 months of age at 5 m.p.i. **e** Comparison of low gamma power in the retrosplenial cortex between of animals seeded at 3 or 6 months of age at 1 and 5 months-post injection. Error bars are standard error of mean. Asterisks indicate significant comparisons (*p* < 0.05). M.p.i refers to months-post injection

Thus, we compared the power spectra values of each frequency band at the 1 and 5 m.p.i timepoints between tau seeded animals and their respective buffer-injected controls. We report significant reductions in low and high gamma power in tau-seeded animals injected at 3 months of age (Fig. 6a, Table 4a). This suggests that the post-injection changes persist and result in a lasting impact on hippocampal gamma function in younger animals. Other power spectra bands did not exhibit significant differences at these timepoints, suggesting that lasting impairment specifically affects gamma oscillations.

**Table 4:** Table of pairwise comparisons of longitudinal phase-amplitude coupling and power spectra differences between buffer and tau-seeded animals: (a) pairwise comparisons of power spectra values of low and high gamma power at 1 and 5 m.p.i. between NL-G-F-tau and NL-G-F-buffer animals at 1 and 5 m.p.i., (b) pairwise comparisons of phase-amplitude coupling of Theta 1-Low Gamma and Theta 2-Low Gamma modulation indices at 5 m.p.i., (c) pairwise comparisons of retrosplenial cortex low gamma power values between NL-G-F-tau and NL-G-F-buffer animals

Frequency band	Pairwise comparison	estimate	SE	df	T ratio	P value
*(a) Post hoc pairwise contrasts of gamma power between NL-G-F-tau and NL-G-F-buffer animals at 1 and 5 m.p.i*						
Low Gamma	3mo NL-G-F-Buffer 1mpi–3mo NL-G-F-Tau 1mpi	2.7109E−01	8.6694E−02	118	3.1270	3.4261E−03
	3mo NL-NL-Buffer 1mpi–3mo NL-NL-Tau 1mpi	3.0630E−01	8.7346E−02	118	3.5068	1.0483E−03
	3mo NL-G-F-Buffer 5mpi–3mo NL-G-F-Tau 5mpi	3.0341E−01	9.8805E−02	118	3.0708	4.0436E−03
	3mo NL-NL-Buffer 5mpi–3mo NL-NL-Tau 5mpi	4.0796E−01	1.0361E−01	118	3.9373	2.4504E−04
	6mo NL-G-F-Buffer 5mpi–6mo NL-G-F-Tau 5mpi	2.9883E−01	1.0656E−01	118	2.8043	8.6269E−03
High Gamma	3mo NL-G-F-Buffer 1mpi–3mo NL-G-F-Tau 1mpi	2.3731E−01	1.0716E−01	116	2.2145	4.7942E−02
	3mo NL-NL-Buffer 1mpi–3mo NL-NL-Tau 1mpi	2.9279E−01	1.1051E−01	116	2.6495	1.6858E−02
	3mo NL-G-F-Buffer 5mpi–3mo NL-G-F-Tau 5mpi	2.9041E−01	1.1581E−01	116	2.5077	2.4054E−02
	3mo NL-NL-Buffer 5mpi–3mo NL-NL-Tau 5mpi	3.2362E−01	1.2555E−01	116	2.5777	2.0239E−02
	6mo NL-NL-Buffer 1mpi–6mo NL-NL-Tau 1mpi	2.8866E−01	1.0728E−01	116	2.6906	1.5172E−02
	6mo NL-G-F-Buffer 5mpi–6mo NL-G-F-Tau 5mpi	2.5549E−01	1.0926E−01	116	2.3384	3.6104E−02
*(b) Post hoc pairwise contrasts of hippocampal phase-amplitude coupling modulation indices between NL-G-F-tau and NL-G-F-buffer animals*						
Theta2-Low Gamma	3 NL-G-F-Buffer 5mpi–3 NL-G-F-Tau 5mpi	6.4002E−05	2.6238E−05	117	2.4393	3.7753E−02
	6 NL-G-F-Buffer 5mpi–6 NL-G-F-Tau 5mpi	6.9312E−05	2.5494E−05	117	2.7187	1.9664E−02
Theta1-Low Gamma	6 NL-G-F-Buffer 5mpi–6 NL-G-F-Tau 5mpi	2.6314E−05	9.8674E−06	117	2.6668	4.0482E−02
*(c) Post hoc pairwise contrasts of retrosplenial cortex low gamma power between NL-G-F-buffer and NL-G-F-tau animals*						
Retrosplenial cortex	6 NL-G-F-buffer 5mpi–6 NL-G-F Tau 5mpi	3.8715E−01	1.0570E−01	118	3.6629	6.2738E−04

Pre refers to pre-injection values, post refers to post-injection values. SE refers to standard error. Df refers to degrees of freedom. Estimate refers to the estimated difference in value between pairwise comparisons

In contrast, in AD-Tau seeded animals injected at 6 months of age, this relationship was not consistently present, suggesting an age effect on tau seeding as well. Significant reductions in both low and high gamma power were noted in NL-G-F-tau mice at 5 m.p.i, and reductions in high gamma power only in NL-tau animals at 1 m.p.i (Fig. 6b, Table 4a), but not at 5 m.p.i. NL-G-F-tau animals seeded at 6 months of age do not exhibit significant differences in gamma power at 1 m.p.i, suggesting that younger animals may be more susceptible to these impairments.

Phase-amplitude coupling differences were also noted in the hippocampus of both age groups at 5 m.p.i, most notably in NL-G-F-tau animals. In NL-G-F-tau animals injected at 6 months, a reduction in both Theta-1 low gamma and Theta-2 low gamma coupling was noted at 5 m.p.i (Fig. 6d, Table 4b) when compared to buffer-injected animals. In contrast, NL-G-F-tau animals injected at 3 months only exhibited differences in Theta-2 low gamma coupling (Fig. 6c, Table 4b), when compared to buffer-injected animals. These results suggest that Theta-gamma hippocampal phase-amplitude coupling may be affected by the interaction between amyloid and tau pathology.

### NL-G-F-Tau animals exhibit deficits in low gamma power spectra in the retrosplenial cortex at 5 m.p.i

Next, we sought to identify if changes were present in other regions of the brain and if brain regions are affected similarly by the presence of either pathology. The interaction effect of Age × Genotype × Treatment × Time post-injection × Brain region on low gamma power indicated that low gamma power was differentially influenced by a combination of both amyloid and tau pathology, which depends on the brain region and is influenced by the amount of time after injection, as well as the age of the animal.

To further understand this interaction effect, we took a closer look at low gamma power across different brain regions, as well as at different ages in seeded and non-seeded animals from both genotypes. We report that only in NL-G-F-tau animals injected at 6 months of age, low gamma power was noted to be significantly reduced in the retrosplenial cortex (Fig. 6e, Table 4c) at 5 m.p.i. This was not noted to be present in NL-NL animals injected with tau, implying an effect only arising from the interaction between amyloid and tau pathology. This was also not noted in younger NL-G-F-tau mice, indicating an age-associated effect, nor at earlier timepoints of recordings, implying that this effect only develops at the later stages. This was also not noted to be significantly different in other brain regions, indicating that impairment of gamma power by these factors is a characteristic specific to the retrosplenial cortex.

## Discussion

In this study, we sought to understand how neurophysiological and histopathological changes associated with pathology evolved in APP-KI animal models.

In terms of histopathological outcomes, the development of AT8-positive tau pathology occurs similarly in terms of the longitudinal progression of tau pathology to previous reports [25]. Tau pathology becomes progressively more abundant with the amount of time after seeding, further providing evidence for the prion-like spreading capabilities of pathological tau [44]. In addition, the presence of amyloid plaque pathology also appears to facilitate the spreading of AT8-positive tau pathology, which is also consistent with the seeding of AD-tau pathology in other animal models of amyloid pathology [25, 45], suggesting no difference regarding this aspect of tau seeding.

We sought to understand if the initial amount of amyloid pathology could be a factor affecting the subsequent development of AT8-positive tau pathology. Our results suggest that this is not the case, as animals injected at 3 or 6 months of age did not show significantly different amounts of AT8-positive tau pathology at 5 m.p.i. A recent paper published by Meisl and colleagues [46], suggests that the doubling of tau, rather than spreading, contributes more to the prevalence of tau pathology after a certain pathological stage. The lack of significant tau pathology difference at 5 m.p.i between animals injected at either 3 or 6 months of age suggests that while amyloid pathology may drive the initial spreading of tau pathology, there may be more than sufficient amyloid plaque pathology in 3-month-old animals to facilitate spreading, and spreading is instead limited by the amount of seeding material. Additionally, subsequent development could become rate-limited by another factor, which could be the rate of pathological tau replication. A longer-term evaluation of tau pathology could reveal if the trajectory of tau pathology development slows down.

Interestingly, the amount of quantified amyloid pathology was noted to be significantly different, depending on both the genotype and the age at which the animal was injected. Notably, the amount of amyloid pathology in the regions distal from the injection site and lacking significant amounts of AT8-positive tau pathology (i.e., Thalamus), did not exhibit a significant difference. NL-G-F animals injected at an older age developed significantly more amyloid pathology after seeding, suggesting that tau seeding induced a greater amount of amyloid pathology in older animals as time progressed. Another study, while also employing injections of seeding material, albeit through a completely different administration route, had shown increases in amyloid pathology following intravenous injections [47], suggested to be linked to neuroinflammation. Further experiments characterizing the status of microglia in regions that exhibit increased or decreased amyloid plaque pathology could shed insight into the role of neuroinflammation altering the presence of amyloid plaque pathology. Due to the effect of age being present in this study, comparing the microglial profile between different animal ages could reveal molecular changes associated with differential outcomes in terms of amyloid plaque pathology.

However, one particular observation of interest was the spatial preference for tau pathology to develop moreso in some regions (i.e., entorhinal cortex and isocortex). than others (i.e., hippocampus). Several reports have indicated one of the earliest regions that exhibit neurofibrillary tangle pathology is the entorhinal cortex [48, 49], but the exact reason why this region is preferred by tau pathology is unclear. One study has suggested that the spatial distribution of 4R tau isoforms is higher in the entorhinal and frontal regions than in the hippocampus of rats [50] and may reflect a similar spatial distribution of tau isoforms in mice. While mice do not exhibit the 3R isoform [51], it is not clear whether the difference in relative levels of the 4R isoforms (0 N, 1 N, 2 N) [52] between regions could influence the development of tau pathology as induced by seeding human-derived tau. Alternatively, region-specific kinase levels involved in the phosphorylation of tau may be another factor[50] that could explain the region-specific difference in AT8 staining as induced via AD-tau seeds.

Another notable outcome was the presence of AT8 pathology localized to the corpus callosum, suggesting a preference for this region. In the primary paper published by He et al. [25], they reported the presence of neuropil threads localized to the corpus callosum, which is likely the case in this study as well. Another study has indicated similar findings [53], and that presence of staining of the corpus callosum could indicate axonal cellular transport of pathogenic tau. A study focusing on the corpus callosum following tau seeding using sarkosyl-insoluble tau noted phospho-tau deposits in oligodendrocytes in the corpus callosum, suggesting that oligodendrocytes may play a role in facilitating tau seeding. Higher magnification microscopy (e.g., cryo EM or confocal microscopy) could provide insight into these questions and a subsequent line of investigation.

Accompanying this finding, older NL-G-F animals also exhibited impaired low gamma power compared to NL-NL animals, particularly in the regions that subsequently exhibit more amyloid pathology (i.e., the retrosplenial cortex and medial entorhinal cortex) even before seeding, suggesting that impairments in gamma oscillations may be correlated with the subsequent exacerbation of amyloid pathology following seeding.

From baseline recordings, a clear reduction of low gamma power was already present across several brain regions in older animals, suggesting a deficit that gradually develops in older NL-G-F animals with the progression of pathology. Other reports have indicated the presence of gamma-related deficiencies in this mouse model [54–57].

Gamma oscillations are believed to facilitate temporal coordination of neural activity and facilitate the coding of information [58], with correlates such as working memory in the hippocampus [59]. The generation of gamma oscillations has been associated with the proper functioning of interneuron populations [60], which have been identified to be impaired in patients suffering from AD [61], as well as several animal models [62, 63], and suggested as the neurophysiological correlate of impaired cognition in humans [64], as well as animal models [65].

With particular relevance to pathology, evidence suggests a role of gamma oscillations in promoting the clearance of AD-associated neuropathology [66, 67], likely via microglial pathways. However, the direction of causality between gamma oscillations and the amount of pathology is not well understood. Our findings of decreased gamma oscillations in older animals, and subsequent exacerbation of amyloid pathology, suggesting that impairments in gamma may be associated with the subsequent development of pathology.

It is still unclear how gamma oscillations may be linked to pathological differences, but microglial function could be a possible mechanism linking this. The presence of amyloid pathology is associated with the activation of microglia [68, 69], whose functional capabilities are also affected by factors such as age [70], and pathological load [71]. The presence of amyloid pathology has also been suggested to prime microglia [72], which is a process that renders microglia prone to an exaggerated inflammatory response following a subsequent inflammatory stimulus [73]. The priming of microglia has been suggested to also correlate with impaired gamma oscillations [74] and may be one cellular correlate of gamma impairments observed in our study. As such, older animals may exhibit more primed microglia due to prolonged exposure to amyloid pathology, and reduced gamma oscillations. The subsequent injection of tau seeding material could elicit an aggravated response from primed microglia, resulting in an exacerbation of amyloid pathology noted in older, tau-seeded animals [75]. Further characterization of microglial response and priming is necessary to understand if the microglial status is indeed related to pathology and gamma oscillation impairments.

In mice injected at a younger age, impairments were noted to be persistent up until at least 5 m.p.i but were not noted to be present in animals injected at an older age. It is unclear why this is the case, but persistent changes noted in younger animals could indicate a disruption in the developmental processes of the brain. Mice have been reported to exhibit maturation of the brain up until 6 months of age [76], with most of the changes occurring within the first 3 months following birth and less prominent changes during the 3 to 6-month window. The impact of tau seeding may have altered the maturation trajectory of animals, resulting in an effect seen in younger but not older animals.

Notably, while NL-tau animals did not show significant increases in AT8-tau pathology in the hippocampus compared to buffer controls nor amyloid pathology, neurophysiological effects were still present. One potential explanation for this could be related to other forms of pathogenic phosphorylated tau, which are not detected using the epitopes recognized by AT8. Additional testing of other phospho-tau antibodies such as pSer396 [77], pSer404 [78] could allow for correlating region-specific neurophysiological changes and phosphorylation states of pathological tau.

When comparing the power spectra of NL-G-F and NL-NL animals, there were no clear differences in power spectra apart from gamma impairments in older NL-G-F animals. Previous reports have indicated that animal models exhibiting amyloid pathology also feature indications of increased neuronal activity [79, 80], broadband increases in power spectra [81, 82], as well as epileptiform activity [80, 81], and EEG alterations [83, 84] that are believed to reflect indications of neuronal hyperexcitability. Neuronal hyperexcitability has been suggested to be detrimental, with links to the development of seizures and epilepsy in AD [85, 86]. However, we note that in our study, APP-KI animals do not appear to exhibit indications of neuronal hyperexcitability in terms of power spectra differences at the ages and times investigated. Other studies using this same animal model have also reported a similar lack of alterations in power spectra using similar modalities [55, 87, 88] when compared to other amyloid mouse models. This suggests that factors other than amyloid plaque pathology could be associated with the presence of hyperexcitability in animal models of amyloid pathology.

Following the injection of AD-Tau seeds into the hippocampus, animals exhibited significant decreases in broadband power spectra, as well as impairments in phase-amplitude coupling. The reductions in power spectra appear to be in line with the currently understood role of tau pathology inducing neuronal silencing [89]. Not much has been characterized about the effects of tau seeding itself on the activity of neurons, but based on the resulting power spectra reductions, could imply some form of neuronal silencing or reduction of excitability likely associated with tau.

The deficits associated with phase-amplitude coupling, being present following injection, as well as at 5 m.p.i suggest an impairment in the hippocampus associated with tau seeding. Phase-amplitude coupling has been suggested to be a mechanism by which integration across populations of neurons occurs [90]. It has also been suggested that this mechanism controls the exchange of information between brain regions by modulating the amplitude of a higher frequency neuronal oscillation to the phase of a lower frequency neuronal oscillation [38]. Deficits in phase-amplitude coupling have also been reported to be present in another study involving tau seeding that persists longitudinally, up to 20 weeks [91]. Our study demonstrates similar impairments but in theta-high gamma coupling at injection, followed by theta-low gamma impairments at later timepoints in the hippocampus, suggesting that the development of tau pathology likely impairs hippocampal phase-amplitude coupling. Notably, this was seen in NL-G-F-tau mice when compared to buffer controls and not in NL-tau mice, indicating a possible interaction effect between amyloid and tau pathology on hippocampal phase-amplitude coupling.

Lastly, NL-G-F-Tau animals injected at 6 months of age exhibited gamma power deficits in the retrosplenial cortex when compared to buffer-injected controls, suggesting that tau pathology may be further impairing gamma oscillations. While this is one possibility, it is hard to disentangle whether the further reduction in gamma power is due to increased amyloid pathology from tau seeding, or the effect of tau itself. Further experiments need to be performed to disentangle the effects of increased amyloid influencing this readout.

However, we note that apart from the more prominent changes in gamma oscillations, a lack of other significant changes in the power spectra, even in the presence of overt amyloid and tau pathology. This is in contrast with several reports of altered power spectra in patients with AD, of which EEG slowing has been suggested to be a prominent feature of AD [92]. A lack of significant increases in lower frequency power such as delta and theta, which may indicate EEG slowing [93], were noted in this present study.

In this study, we also chose to apply the neurophysiological readout of waveform complexity in the form of the HFD score, a non-linear approach to measuring the self-similarity of the LFP across multiple timescales [39]. This has been used to evaluate the presence of AD in clinical settings [93, 94] and was intended to provide a potentially clinically relevant readout of neurophysiology. However, this endpoint did not exhibit significant interactions with the factors tested. Complexity scores have also been suggested to accompany the slowing of the EEG, which was also not noted in this study [93], and could be a possible explanation for the lack of HFD score changes.

When interpreting these findings of this study in the context of the pathophysiological progression of AD, we suggest that several aspects and limitations should be considered. Primarily, this study involves the injection of AD-tau seeding material derived from patient brain samples, which may induce a more physiologically similar form of tau pathology compared to other methods such as transgenic methods [21]. However, it must be noted that the process of injecting seeding material is by no means physiologically representative of the disease. In addition, the buffer control solutions used in this study may not serve as the best control for tau seeding injections due to the presence of other molecules apart from tau in the seeding material that may influence neurophysiological outcomes. Ideally, a control solution derived from a pool of healthy, age-matched patients would be control for these factors, but may not be entirely feasible. Secondly, the time scale of these experiments was designed with detecting neurophysiological changes at the earlier stages of AD in mind, where therapeutic intervention is believed to hold more promise than at the later stages of the disease. It has been suggested that neurophysiological manifestations of AD tend to be more prominent towards the later stages of the disease [95] and that more subtle changes occur at the earlier phases of the disease, which may be much harder to detect. Thus, it may be possible that even after 5 months after injection, the lack of overt detectable neurophysiological changes could be due to a lack of pathological severity. This does not imply that the readouts are irrelevant for diagnosis but reiterates the difficulty associated with finding robust neurophysiological readouts that accompany changes associated with AD at the earlier stages of the disease.

## Conclusion

In this present study, we have characterized both the pathological and neurophysiological implications of seeding tau pathology in the APP-KI animal model with and without amyloid pathology, that does not exhibit APP overexpression. Amyloid pathology serves to facilitate the spread of tau pathology but also appears to be exacerbated by the presence of tau pathology, highlighting how these pathologies synergize and interact. The presence of impaired gamma oscillations preceding exacerbation of amyloid pathology may already indicate underlying factors that contribute to the further development of pathology. In addition, the seeding and development of tau pathology result in immediate and lasting impairments in power spectra and phase-amplitude coupling, which appear to be affected by the age at which the animal was seeded. We also did not note changes in power spectra that may be indicative of neuronal hyperexcitability, suggesting that factors other than amyloid plaque pathology may be contributing to network hyperexcitability in animal models of amyloid pathology. Our study attempts to shed light on neurophysiological changes associated with the development of pathology while controlling for some confounds related to current animal models. These findings bring us closer to identifying early neurophysiological changes associated with the development of pathology, and a potential neurophysiological indicator for the risk of subsequently increased amyloid pathology.

## Supplementary Information

**Additional file 1.** Supplementary document containing additional information on the Methods, Non-identifiable patient characteristics, Quality control data, as well as non-significant findings.

## Data Availability

The datasets used and/or analyzed during the current study are available from the corresponding author on reasonable request.

## Abbreviations

AD: Alzheimer's Disease
AICD: APP Intracellular Domain
AP: Anterior–posterior
APP: Amyloid Precursoe Protein
APP-KI: App Knock In
CA1: Cornu Ammonis Region 1
DV: Dorso-ventral
EEG: Electroencephalogrphy
FDR: False Discovery Date
GLMM: General Linear Mixed Model
hAPP: Human Amyloid Precursor Protein
HFD: Higuchi Fractal Dimension
LFP: Local field potential
LTP: Long-term potentiation
MAPT: Microtubule associated protein Tau
MI: Modulation Index
ML: Medial–Lateral
NFT: Neurofibrillary Tangles
PBS: Phosphate-buffered saline
PCR: Polymerase chain reaction
PFA: Paraformaldehyde
PFTAA: Pentameric formyl thiophene acetic acid
