# Supplementary material for: Pathological and neurophysiological outcomes of seeding human-derived tau pathology in the APP-KI NL-G-F and NL-NL mouse models of Alzheimer’s Disease
Source: Acta Neuropathol Commun. 2022 Jun 23;10:92. doi: 10.1186/s40478-022-01393-w (PMC9219251; doi:10.1186/s40478-022-01393-w)
Supplement: Supplementary file 1 — Additional file 1. Supplementary document containing additional information on the Methods, Non-identifiable patient characteristics, Quality control data, as well as non-significant findings. [file 40478_2022_1393_MOESM1_ESM.docx]

Supplementary information

# Supplementary Methods

## M1. Electrolesion parameters and settings

The stimulation for electrolesion was a rectangular AC pulse signal with a duty cycle of 25%, at a current of ±250µA for 150 cycles at 2Hz. The stimulator ground wire was connected to ground electrode of the animal and stimulation wire was connected to each recording and reference electrode on the animal.

## M2. DeepLabCut model construction and activity level estimation

Video data obtained during recording was cropped and downscaled using ffmpeg version 4.2 via a custom MATLAB script. Videos were cropped to a relative rectangular frame that fit the dimensions of the home cage in the recording. These videos were subsequently resized to 50% of the cropped width and height.

Analysis of the videos was carried out using DeepLabCut to estimate the position of body parts of the animal, and subsequently, the activity of the animal. A DeepLabCut model was manually trained to track the head, body and base of the tail using 25 different animals across multiple recording boxes in order to build a generalizable neural network model. 20 still frames from each video recording for a total of 500 frames were extracted using the kmeans algorithm for frame selection and body parts of the animal were manually labelled by the experimenter where possible. If the animal was not visible due to visual obstruction by the environment, the respective tracking point was removed from that frame. The base neural network model was selected as resnet_50 and was trained using the default augmentation method and frame dataset specified above. The model was trained for up to 500000 iterations, or until the loss plateaued. Once the network was trained, the network was evaluated on naïve video data not used in the training or testing process to evaluate the generalizability of the network and detect tracking errors. Outlier frames were extracted to retrain the model for 2 more iterations using the jump algorithm specified in the software package.

The position data from the animal was generated for each frame of the video and motion data was calculated from frame position data interpolation. Motion data was smoothed across 25 frames using mean averaging, and additional movement artefacts were detected using a standard deviation filter of 5 SD and removed by interpolating the position data. The classification of animal activity was a binary state of 0 (inactive) or 1 (active) with an activity threshold calculated from 30% of the peak motion displacement over the entire recording session

## M3. Artefact and noise removal algorithms and specific exclusion criteria.

Local field potential data acquired from recordings was 1Hz high-pass filtered using a 2^nd^ order Butterworth zero-phase IIR software filter constructed using the Signal Processing Toolbox in MATLAB 2016a. Signal quality was inspected at multiple levels to remove noise and artefacts by excluding animals that did not meet the quality criteria. Signals were visually inspected for the presence of 50Hz noise, in one (indicative of a broken electrode) or multiple recording channels (indicative of multiple damaged electrodes or broken ground electrode) and electrodes were excluded from subsequent analysis. The presence of high-amplitude low frequency signals slightly above 1Hz correlated with the movement of the animal was indicative of a broken reference electrode and recordings containing that artefact were excluded from the analysis. Subsequent artefact detection involved the removal of non-physiological high-amplitude spikes that had similar amplitudes across all channels using a standard deviation cutoff of 10 SD using a custom in-house MATLAB script. Epochs The final quality check was carried out using histological verification of electrode positions in the brain regions via the electro-lesion procedure described above. Electrode locations were checked in a subset of animals to verify the accuracy of electrodes.

## M4. General linear mixed model and statistical analysis for histological readouts

A general linear mixed model (GLMM) was fit to the amount of AT8, amyloid or colocalized pathology with Sex, Age at injection, Genotype, Treatment (seeded vs. buffer), Time post injection and Brain region as fixed effects (main effects), and the Age x Genotype x Treatment x Time post injection x Brain region as interaction term (including all lower-order interactions that constitute this 5th-order interaction). A random intercept was included for each animal. A backward stepwise elimination model building procedure was used in which a series of likelihood ratio tests were conducted to evaluate the statistical significance of the fixed effects (starting with the highest-order terms in a hierarchical way). The asymptotic null distribution of the likelihood ratio test is a $\chi^{2}$ with degrees of freedom equal to the difference in the number of fixed-effect parameters in the models that are being compared (Verbeke & Molenberghs, 2000). An alpha-level of 0.05 was used throughout the analyses, using Benjamini-Hochberg false discovery rate to account for multiple testing. Marginal residuals were visually inspected to check the normality assumption, and homoscedasticity was evaluated based on graphical inspection of the marginal residuals against the predicted values. There were no major violation of the model assumptions.

The dependent variables of PFTAA (i.e. amyloid pathology) and AT8 (tau pathology), or colocalized pathology (i.e. plaque-associated tau) was fit using the independent variables of sex (binary, categorical), genotype (binary, categorical), treatment (binary, categorical), brain region (nominal, categorical), time post injection (numerical, continuous) and age (numerical, continuous) as main effects and the genotype x treatment x brain region x time post injection x age interaction term (including all lower-order interaction terms). The model included a random intercept for animal. The model can be written as:

$Y_{is}=\beta_{0}+b_{i}+\beta_{1}{Gender}_{i}+\beta_{2}{Age}_{i}+\beta_{3}{Brain region}_{i}+\beta_{4}{Genotype}_{i}+\beta_{5}{Treatment}_{i}+\beta_{6}{Time}_{i}+\ldots+ \beta_{k}\left( {Age}_{i}*{Brain region}_{i}*{Genotype}_{i}*{Treatment}_{i}*{Time}_{i} \right)+\varepsilon_{is}$,

With:

$Y_{is}$= the measured amyloid or tau pathology for the *i*-th animal in the *s*-th brain region

$\beta_{0}$= the intercept

$b_{i}$= the random intercept for the *i*-th animal

$\beta_{1}$ to $\beta_{k}$ = the fixed effects for the main effects and interaction terms

Notice that shorthand notation is used to avoid a very long formula, i.e., … refers to all lower-order interaction terms of the ${Age}_{i}*{Brain region}_{i}*{Genotype}_{i}*{Treatment}_{i}*{Time}_{i}$ interaction term in the model. In addition, brain region is a categorical variable with 4 levels so it is coded using three dummy variables in the mixed model (i.e., the main effect of brain region is in fact captured by 3 fixed effects (beta’s) instead of only one as is shown in the above equation for brevity). The model assumes that the residuals and the random intercepts are normally distributed with mean zero and variance sigma, i.e., $\varepsilon_{is}\sim N(0, \sigma_{\varepsilon})$ and $b_{i}\sim N(0, \sigma_{b})$.

The following R code is used to fit the model as follows:

M1 <- lme (fixed = Pathology~SEX+Age*BrainRegion*GENOTYPE*TREAT*TP, data=Data_filt, random = ~1|MouseID, method="ML", na.action = na.omit)

Whereby Pathology refers to the quantified amount of amyloid, tau or colocalized pathology, SEX refers to the sex of the animal, Age refers to the age of the animal when it was injected (i.e. 3 or 6 months of age), BrainRegion refers to the respective brain region (Hippocampal region, Entorhinal area, Isocortex or Thalamus), GENOTYPE refers to the genotype of the animal (TG or WT), treatment refers to the type of injection (i.e. buffer or tau-seed) and TP refers to the amount of time after injection (1, 3, or 5 months post injection). Data refers to the dataframe used for analysis (amyloid or tau data values). The random intercepts are specified with the random = ~1|MouseID option. The method for fitting the model is specified as method="ML", where ML refers to maximum likelihood. na.action = na.omit specifies that the model omits data that contains missing values.

Subsequently, the interaction effect was tested by comparing to a model that does not contain the interaction term of interest using the anova package. This is described in the code as such:

M1b<- lme(fixed= LabelRatio~SEX+TP*TREAT*GENOTYPE*LabelName+TP*TREAT*GENOTYPE*Age+TP*TREAT*LabelName*Age+TP*GENOTYPE*LabelName*Age+TREAT*GENOTYPE*LabelName*Age+TP*TREAT*GENOTYPE+TP*TREAT*LabelName+TP*GENOTYPE*LabelName+TREAT*GENOTYPE*LabelName+TP*TREAT*Age+TP*GENOTYPE*Age+TREAT*GENOTYPE*Age+TP*LabelName*Age+TREAT*LabelName*Age+GENOTYPE*LabelName*Age+TP*TREAT+TP*GENOTYPE+TREAT*GENOTYPE+TP*LabelName+TREAT*LabelName+GENOTYPE*LabelName+TP*Age+TREAT*Age+GENOTYPE*Age+LabelName*Age, data=Data _filt, random = ~1|MouseID, method="ML", na.action = na.omit)

anova(M1,M1b)

Where M1b refers to the model containing all interaction terms except for the interaction term of interest (i.e. Age*LabelName*GENOTYPE*TREAT*TP).

The anova command compared the two models and generates a likelihood ratio between the two models, as well as a p-value indicating if the two models differ significantly, and if the model containing the interaction term fits the data better.

## M5. General linear model for analysis of neurophysiological readouts

Similar to the model described in M4., the neurophysiological outcomes were evaluated using the general linear model approach. The dependent variables of power spectra (delta, theta 1, theta 2, low gamma, high gamma), phase amplitude coupling (Theta1-Low gamma, Theta2-Low gamma, Theta1-High gamma, Theta2-High gamma) and Higuchi Fractal Dimension score were fit using the using the independent variables of sex (binary, categorical), and an interaction term of genotype (binary, categorical), treatment (binary, categorical), electrode (nominal, categorical), time post injection (numerical, continuous) and age (numerical, continuous) as fixed effects. The model can be written as:

$Y_{isj}=\beta_{0}+b_{i}+\beta_{1}{Gender}_{i}+\beta_{2}{Age}_{i}+\beta_{3}{Brain region}_{i}+\beta_{4}{Genotype}_{i}+\beta_{5}{Treatment}_{i}+\beta_{6}{Time}_{i}+\ldots+ \beta_{k}\left( {Age}_{i}*{Brain region}_{i}*{Genotype}_{i}*{Treatment}_{i}*{Time}_{i} \right)+\varepsilon_{isj}$,

With:

$Y_{isj}$= the measured amyloid or tau pathology for the *i*-th animal in the *s*-th brain region of the j-th timepoint

$\beta_{0}$= the intercept

$b_{i}$= the random intercept for the *i*-th animal

$\beta_{1}$ to $\beta_{k}$ = the fixed effects for the main effects and interaction terms

Notice that shorthand notation is used to avoid a very long formula, i.e., … refers to all lower-order interaction terms of the ${Age}_{i}*{Brain region}_{i}*{Genotype}_{i}*{Treatment}_{i}*{Time}_{i}$ interaction term in the model. In addition, brain region is a categorical variable with 4 levels so it is coded using three dummy variables in the mixed model (i.e., the main effect of brain region is in fact captured by 3 fixed effects (beta’s) instead of only one as is shown in the above equation for brevity). The model assumes that the residuals and the random intercepts are normally distributed with mean zero and variance sigma, i.e., $\varepsilon_{isj}\sim N(0, \sigma_{\varepsilon})$ and $b_{i}\sim N(0, \sigma_{b})$.

The code describing the model is as follows:

M1 <- lme(fixed = THETA_1~SEX+Age*ELECTRODE*GENOTYPE*TREAT*TP, data=Data, random = ~1|ANIM_REF, method="ML", na.action = na.omit)

Whereby M1 refers to the fitted model, THETA_1 in this instance, refers to the power spectra values of the theta 1 frequency band, SEX refers to the sex of the animal, Age refers to the age of the animal when it was injected (i.e. 3 or 6 months of age), ELECTRODE refers to the respective electrode as a proxy for brain region (Hippocampal CA1 region, Entorhinal cortex, Isocortex or Thalamus), GENOTYPE refers to the genotype of the animal (TG or WT), treatment refers to the type of injection (i.e. buffer or tau-seed) and TP refers to the amount of time after injection (1, 3, or 5 months post injection). Data refers to the dataframe used for analysis (i.e. power spectra, HFD scores, phase-amplitude coupling values etc.). The random intercepts are specified with the random = ~1|ANIM_REF option. The method for fitting the model is specified as method="ML", where ML refers to maximum likelihood. na.action = na.omit specifies that the model omits data that contains missing values.

Subsequently, the interaction effect was tested by comparing to a model that does not contain the interaction term of interest using the anova package. This is described in the code as such:

M1b<-lme(fixed = THETA_1~SEX+Age*ELECTRODE*GENOTYPE*TREAT+Age*ELECTRODE*GENOTYPE*TP+Age*ELECTRODE*TREAT*TP+Age*GENOTYPE*TREAT*TP+ELECTRODE*GENOTYPE*TREAT*TP+Age*ELECTRODE*GENOTYPE+Age*ELECTRODE*TREAT+Age*ELECTRODE*TP+Age*GENOTYPE*TREAT+Age*GENOTYPE*TP+Age*TREAT*TP+ELECTRODE*GENOTYPE*TREAT+ELECTRODE*GENOTYPE*TP+ELECTRODE*TREAT*TP+GENOTYPE*TREAT*TP+Age*ELECTRODE+Age*GENOTYPE+Age*TREAT+Age*TP+ELECTRODE*GENOTYPE+ELECTRODE*TREAT+ELECTRODE*TP+GENOTYPE*TREAT+GENOTYPE*TP*+TREAT*TP,
data=Data, random = ~1|ANIM_REF, method="ML", na.action = na.omit)

anova(M1,M1b)

Where M1b refers to the model containing all interaction terms except for the interaction term of interest (i.e. Age*LabelName*GENOTYPE*TREAT*TP).

Similarly, for electrophysiological analyses general linear mixed model (GLMM) was fit to the neurophysiological readouts with Sex, Age at injection, Genotype, Treatment (seeded vs. buffer), Time post injection and Brain region as fixed effects (main effects), and the Age x Genotype x Treatment x Time post injection x Brain region as interaction term (including all lower-order interactions that constitute this 5th-order interaction).

# Supplementary Figures

## Supplementary Figure 1
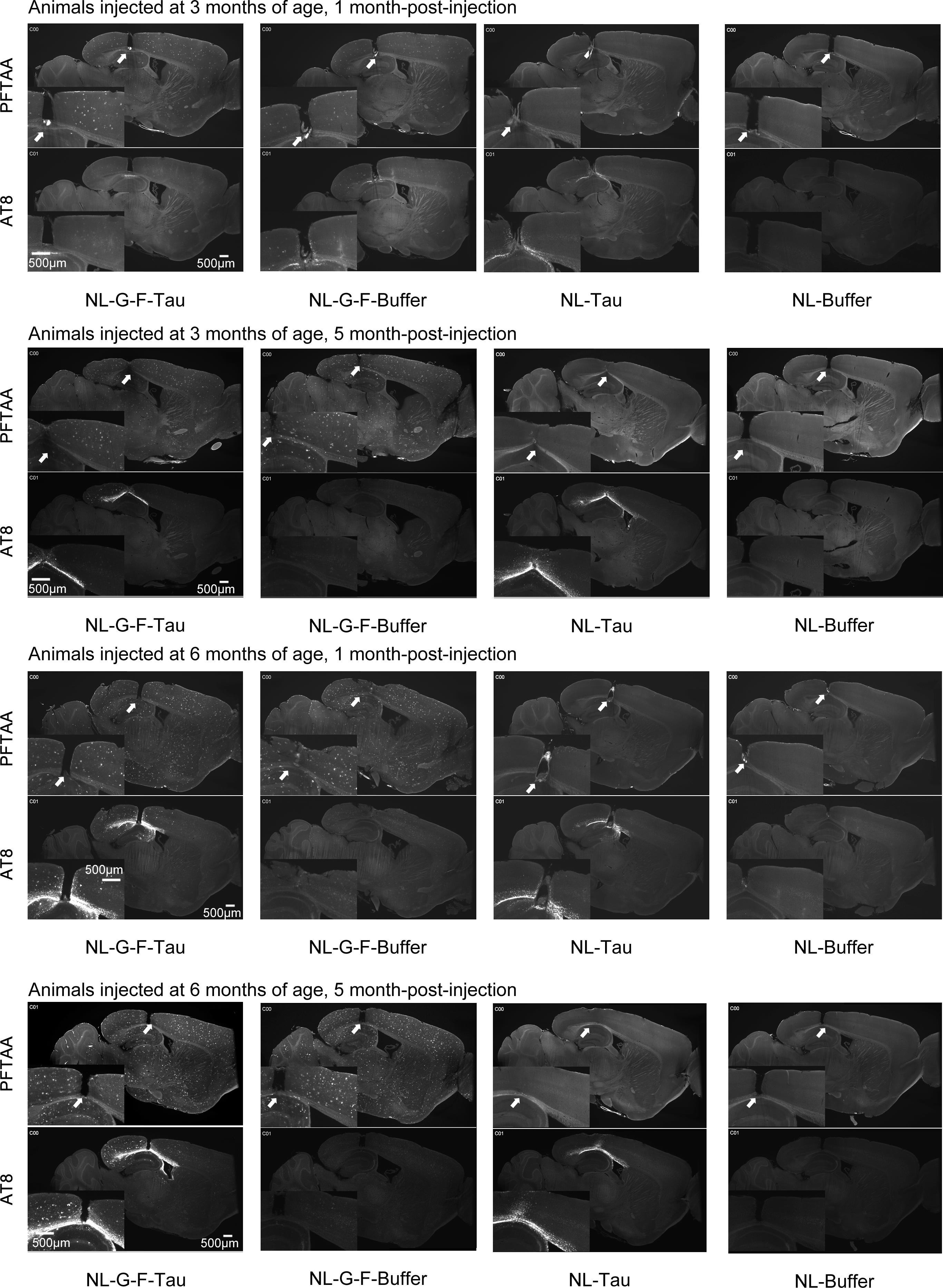


## Supplementary Figure 2


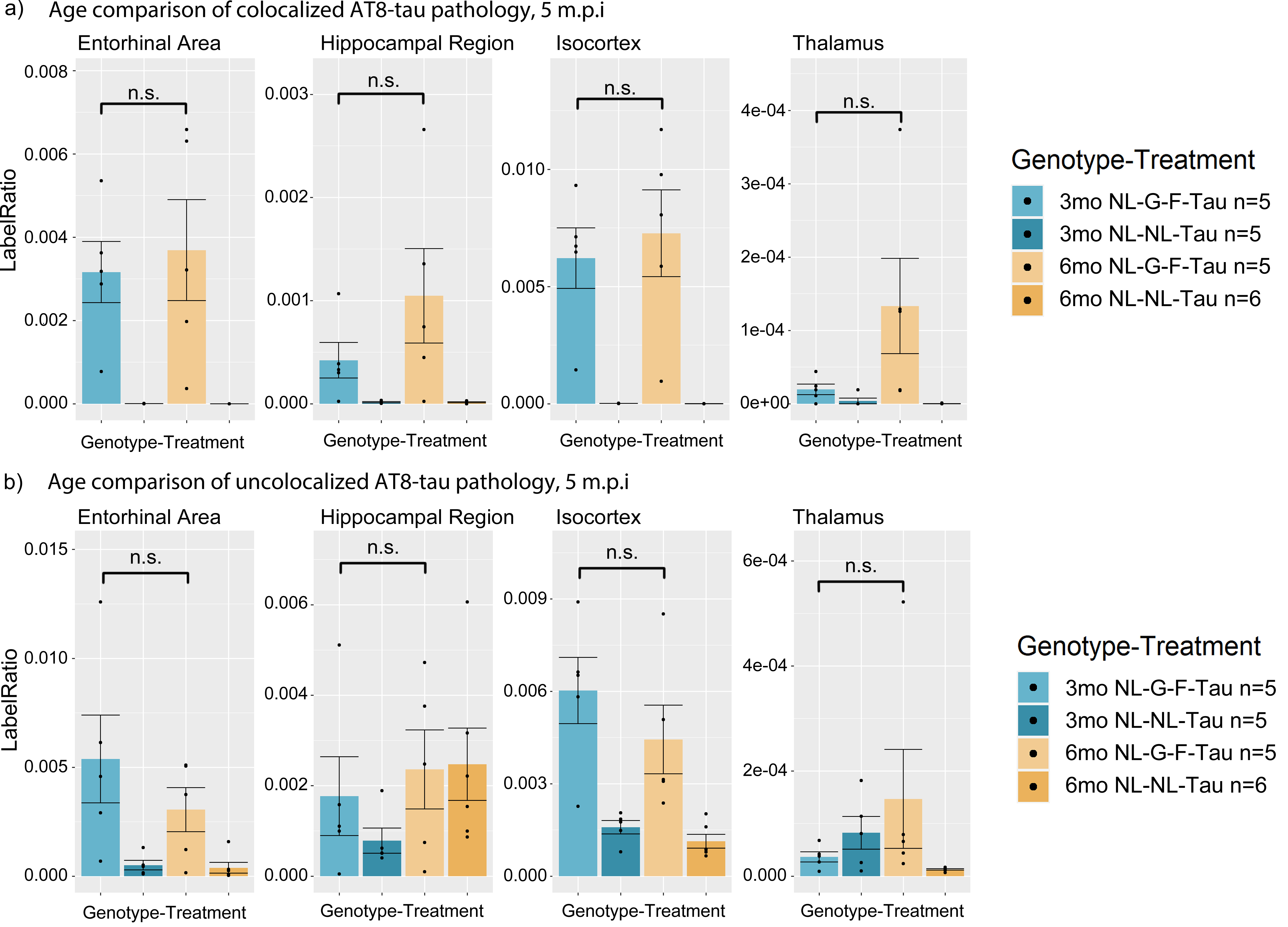


## Supplementary Figure 3
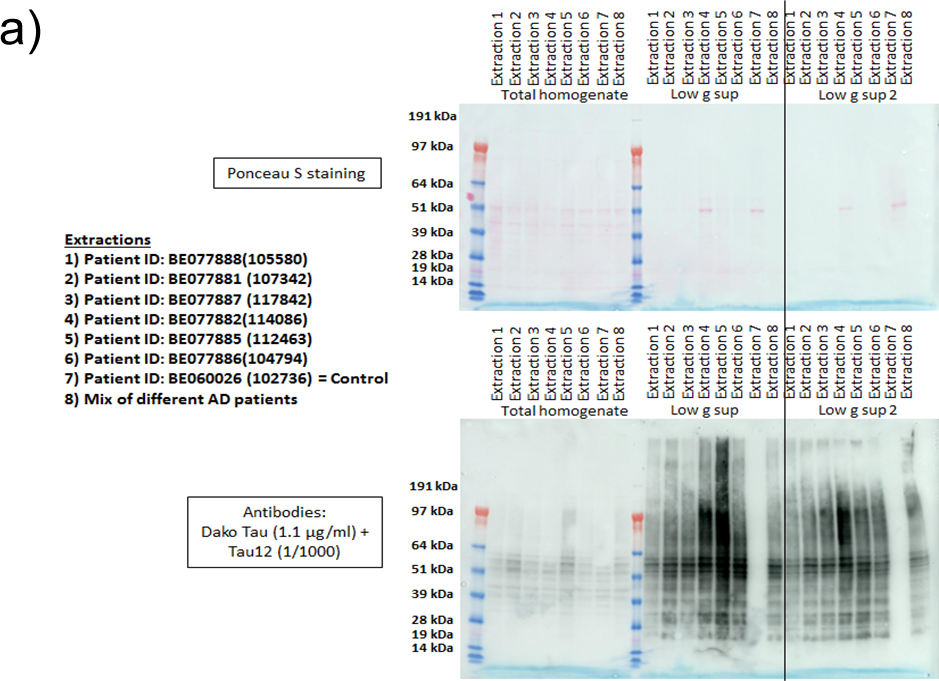


## Supplementary Figure 4


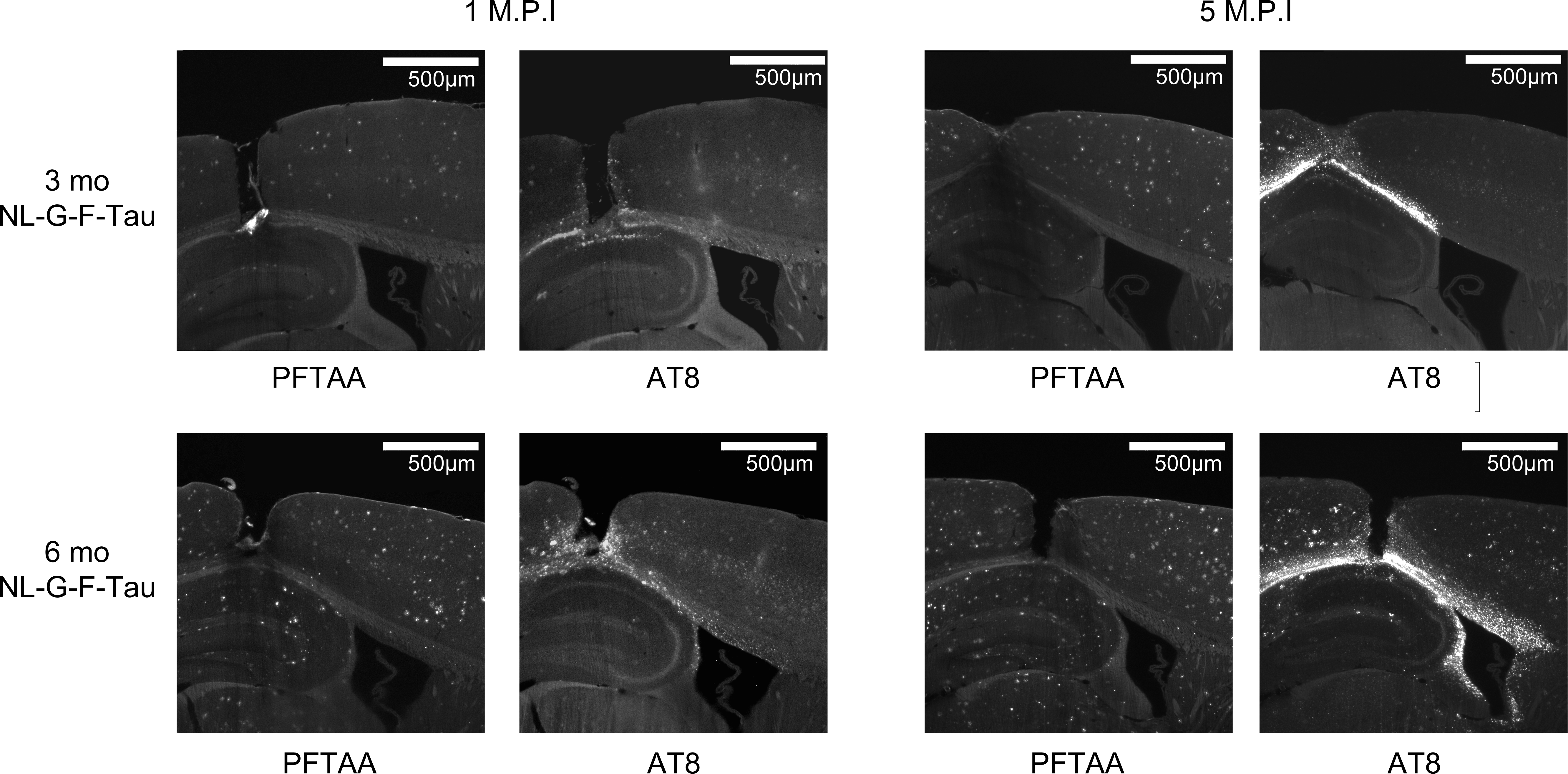


# Supplementary Figure Legends

## Supplementary Figure 1

Representative light sheet images from 3- and 6-month-old animals at 1 and 5 months-post injection, showing development of AT8-positive tau pathology in NL-tau and NL-G-F-Tau mice, but not in NL-G-F-Buffer nor NL-Buffer. Images also show that NL-G-F animals exhibit amyloid pathology as visualised with PFTAA, but NL-NL animals do not show any pathology at these timepoints and ages. The lack of PFTAA signal in NL-Tau serves to indicate that PFTAA under these settings do not detect AT8-positive tau pathology, and allows for easily distinguishing tau and amyloid pathology. White arrows indicate the site of injection (i.e., the hippocampus).

## Supplementary Figure 2

Quantification of amyloid pathology, as well as colocalized and non-colocalized AT8-tau pathology at 5 months-post-injection. Comparison of a) colocalized and b) non-colocalized AT8 tau pathology between animals injected at 3-months-old and 6-months-old at 5 months-post-injection. Error bars are standard error of mean. Asterisks indicate significant comparisons (p<0.05). n.s. refers to non-significant comparisons. M.p.i refers to months-post injection.

## Supplementary Figure 3

Western blotting and Ponceau S staining using Tau antibodies. Tau seeding material was prepared from 6 different patients and combined to form a pool of seeds. Patient 7 served as control for the tau extraction protocol, showing no tau staining in either supernatant after purification. Western blotting was done on total brain homogenate, and supernatant after 2 rounds of centrifugation.

## Supplementary Figure 4

Higher magnification images of histological images seen in Fig. 1. Images serve to highlight the longitudinal development of AT8-positive tau pathology from 1 to 5 moths post injection. M.p.i refers to months post injection. Mo refers to months old.

# Supplementary Tables

## Supplementary Table 1

| Patient Data |  |  |  |  |  |  |  |  |  |  |  |  |  |
| --- | --- | --- | --- | --- | --- | --- | --- | --- | --- | --- | --- | --- | --- |
| Patient ID | Age at Death | PMI | Sex | Clinical Diagnosis | Race | Ethnicity | Global Age Onset | APOE | MF Tau | MF Thio Plaques | MF Antibody Plaques | MF aSyn | MF TDP43 |
| BE077883 | 72 | 12 | Female | FTLD-NOS | White | Non-Latino | 58 | E3/E3 | 2+ | 2+ | 3+ | 0 | 0 |
| BE077881 | 71 | 4.5 | Female | PPA (Logopenic) | White | Non-Latino | 62 | E2/E4 | 3+ | 3+ |  | 0 | 0 |
| BE077890 | 79 | 4 | Female | Alzheimer's Disease Probable | Black | Non-Latino | 69 | E3/E4 | 3+ | 2+ | 3+ | 0 | 0 |
| BE077885 | 73 | 4 | Female | PPA (Semantic dementia) | White | Non-Latino | 59 | E3/E3 | 3+ | 3+ |  | 0 | 0 |
| BE077889 | 78 | 10 | Female | Alzheimer's Disease Probable | Black | Non-Latino | 62 | E4/E4 | 3+ | 3+ |  |  |  |
| BE077886 | 74 | 4 | Female | Alzheimer's Disease Probable | White | Non-Latino | 62 | E4/E4 | 3+ | 2+ | 3+ | 1+ | 0 |
| Sample Data |  |  |  |  |  |  |  |  |  |  |  |  |  |
| Patient sample | Protein concentration (mg/ml) |  |  |  |  |  |  |  |  |  |  |  |  |
| BE077883 | 1.760893575 |  |  |  |  |  |  |  |  |  |  |  |  |
| BE077881 | 1.977165486 |  |  |  |  |  |  |  |  |  |  |  |  |
| BE077890 | 2.112351699 |  |  |  |  |  |  |  |  |  |  |  |  |
| BE077885 | 5.035435237 |  |  |  |  |  |  |  |  |  |  |  |  |
| BE077889 | 4.683272218 |  |  |  |  |  |  |  |  |  |  |  |  |
| BE077886 | 3.410776788 |  |  |  |  |  |  |  |  |  |  |  |  |
| Pooled Sample | 2.572078482 |  |  |  |  |  |  |  |  |  |  |  |  |

## Supplementary Table 2

| a) Post hoc pairwise contrasts of colocalized AT8-positive tau pathology between 3 and 6mo NL-G-F-tau animals | |  |  |  |  |  |
| --- | --- | --- | --- | --- | --- | --- |
| Brain region | Pairwise comparison | estimate | SE | df | T ratio | P value |
| Entorhinal Area | 3mo NL-G-F-Tau 5mpi - 6mo NL-G-F-Tau 5mpi | -0.0005 | 0.0005 | 69 | -0.9725 | 1.0000E+00 |
|  |  |  |  |  |  |  |
| Isocortex | 3mo NL-G-F-Tau 5mpi - 6mo NL-G-F-Tau 5mpi | -0.0011 | 0.0005 | 69 | -1.9531 | 2.8361E-01 |
|  |  |  |  |  |  |  |
| Hippocampal formation | 3mo NL-G-F-Tau 5mpi - 6mo NL-G-F-Tau 5mpi | -0.0006 | 0.0005 | 69 | -1.1522 | 1.0000E+00 |
|  |  |  |  |  |  |  |
| Thalamus | 3mo NL-G-F-Tau 5mpi - 6mo NL-G-F-Tau 5mpi | -0.0001 | 0.0005 | 69 | -0.2095 | 1.0000E+00 |
|  |  |  |  |  |  |  |
| b) Post hoc pairwise contrasts of non-colocalized AT8-positive tau pathology between 3 and 6mo NL-G-F-tau animals | |  |  |  |  |  |
| Brain region | Pairwise comparison | estimate | SE | df | T ratio | P value |
| Entorhinal Area | 3mo NL-G-F-Tau 5mpi - 6mo NL-G-F-Tau 5mpi | 0.0008 | 0.0009 | 70 | 0.9418 | 9.4138E-01 |
|  |  |  |  |  |  |  |
| Isocortex | 3mo NL-G-F-Tau 5mpi - 6mo NL-G-F-Tau 5mpi | 0.0002 | 0.0009 | 70 | 0.1806 | 9.9894E-01 |
|  |  |  |  |  |  |  |
| Hippocampal formation | 3mo NL-G-F-Tau 5mpi - 6mo NL-G-F-Tau 5mpi | -0.0013 | 0.0009 | 70 | -1.4039 | 5.1982E-01 |
|  |  |  |  |  |  |  |
| Thalamus | 3mo NL-G-F-Tau 5mpi - 6mo NL-G-F-Tau 5mpi | 0.0003 | 0.0009 | 70 | 0.3389 | 9.9894E-01 |
|  |  |  |  |  |  |  |

# Supplementary Table Legends

## Supplementary Table 1 Legend

Table containing patient information, describing the age, post-mortem interval (PMI), Sex, clinical diagnosis, Race, Ethnicity, Age of Onset, Presence of APOE mutations, presence of Tau, Thioflavin-positive plaques, Antibody-positive plaques, alpha-synuclein and TDP43. Data regarding the patient-specific total protein concentrations of the seeding material, following purification, and the protein concentration after pooling.

## Supplementary Table 2 Legend

Table containing pairwise comparisons of colocalized and non-colocalized AT8-positive tau pathology. b) Comparisons of colocalized AT8-positive tau pathology between NL-G-F-tau and NL-G-F-buffer animals at 5 m.p.i. c) Comparisons of non-colocalized AT8-positive tau pathology between NL-G-F-tau and NL-G-F-buffer animals at 5 m.p.i. SE refers to standard error. Df refers to degrees of freedom. Estimate refers to the estimated difference in value between pairwise comparisons. mo refers to months-old at injection.
